# Energy solutions of singular SPDEs on Hilbert spaces with applications to domains with boundary conditions

**DOI:** 10.1007/s40072-026-00424-0

**Published:** 2026-04-04

**Authors:** Lukas Gräfner, Nicolas Perkowski, Shyam Popat

**Affiliations:** 1 Department of Statistics, University of Warwick, Coventry, CV4 7AL UK; 2 Institut für Mathematik, Freie Universität Berlin, Arnimallee 7, 14195 Berlin, Germany; 3 Centre de Mathématiques Appliquées (CMAP), École Polytechnique, 91120 Palaiseau, France

**Keywords:** Energy solutions, boundary conditions, Gelfand triple, stochastic Burgers equation, 60H17, 60H07, 60H50

## Abstract

In this paper we extend the theory of energy solutions for singular SPDEs, focusing on equations driven by highly irregular noise with bilinear nonlinearities, including scaling critical examples. By introducing Gelfand triples and leveraging infinite-dimensional analysis in Hilbert spaces together with an integration by parts formula under the invariant measure, we largely eliminate the need for Fourier series and chaos expansions. This approach broadens the applicability of energy solutions to a wider class of SPDEs, offering a unified treatment of various domains and boundary conditions. Our examples are motivated by recent work on scaling limits of interacting particle systems.

## Introduction

The semigroup approach to SPDEs in Hilbert spaces, as developed in the seminal monograph by Da Prato and Zabczyk [11] is based on infinite-dimensional analysis in Hilbert spaces combined with stochastic analysis and martingale techniques. It offers a unified approach to a wide range of equations driven by infinite-dimensional Wiener processes. The semigroup approach is notable for its versatility, addressing not only well-posedness but also more advanced aspects including martingale problems and connections to infinite-dimensional Kolmogorov equations [4, 10], study of invariant measures and ergodicity [9], stability and dynamic properties, control theory, numerical analysis, regularization by noise, reflected equations, or scaling limits. This generality has made the semigroup approach an invaluable tool for researchers tackling both theoretical and applied problems in infinite-dimensional stochastic systems.

However, for certain classes of SPDEs, particularly those involving highly singular noise and nonlinearities, the traditional semigroup approach breaks down. When the noise becomes too irregular, the solution may no longer take values in a space on which the nonlinearity is well-defined. In this case, renormalization becomes essential, and a new conceptual framework is required to handle the resulting singularities. This gap has been filled by the development of singular SPDE theory, as pioneered by Hairer’s theory of regularity structures [27] and Gubinelli-Imkeller-Perkowski’s paracontrolled calculus [16], both originating in Lyons’s rough path theory [30]. These pathwise techniques introduced the idea of scaling subcriticality, where the nonlinearities become small at small scales. This enables a perturbative expansion of the solution around the linearized equation that gains regularity, in the spirit of previous works by Da Prato and Debussche [3, 5]. Singular SPDEs grew into a vibrant new area of research in SPDEs. But despite the impressive progress in the last decade, the probabilistic structure of singular SPDEs is still worse understood than for SPDEs on Hilbert spaces in the semigroup approach. In particular, the pathwise nature of these methods complicates the study of martingale properties or the relation to infinite-dimensional Kolmogorov equations, and the connection to the broader probabilistic methods used in the semigroup approach is largely absent in the existing theories of singular SPDEs.

This is where energy solutions become relevant. Energy solutions offer a probabilistic alternative for certain singular SPDEs with a tractable probabilistic structure. They provide a martingale-based theory that is compatible with Itô calculus. Energy solutions were first defined and constructed by Gonçalves and Jara [18] for the stochastic Burgers equation in the context of scaling limits for particle systems. Gubinelli and Jara [17] found a slightly stronger formulation and extended the construction to stochastic Navier-Stokes equations and fractional Burgers equations. In both of these works, the question of uniqueness remained open. For the stochastic Burgers equation, Gubinelli and Perkowski [20] later proved well-posedness with the help of the Cole-Hopf transform that linearizes the equation. In a subsequent work [21] they extended the well-posedness theory to other equations including the fractional stochastic Burgers equation in the entire subcritical regime, by leveraging a martingale problem approach. Notably, they constructed the infinitesimal generator and solved the infinite-dimensional Kolmogorov backward equation by combining Gaussian analysis with Fourier series computations. More recently, the ongoing Ph.D. thesis of Gräfner [24] has advanced the theory of energy solutions by adopting a more systematic functional analytic approach that largely removes the need for Fourier series computations. This approach relies on the chaos expansion under a Gaussian or non-Gaussian reference measure, and it is able to handle scaling critical equations like the critical fractional stochastic Burgers equation and the stochastic surface quasi-geostrophic equation. It also allows for non-stationary perturbations of drift and diffusion, time-dependent coefficients, and it provides a PDE approach (compared to the semigroup approach used here) for the Kolmogorov backward equation, as well as Markov selection results for some scaling super-critical equations.

Our current work strengthens the theory of energy solutions, broadening its scope and enhancing its robustness. We build upon the foundational ideas of SPDEs on Hilbert spaces [11] and combine them with the Gelfand triples from the variational approach to SPDEs [29] to rigorously define the operators involved in the stochastic dynamics, also inspired by the construction of singular operators like the Anderson Hamiltonian in the context of singular SPDEs [1]. We consider densely embedded Hilbert spaces $V\subset H \subset V^*$ and our focus is on equations of the form

1 $$\begin{aligned} \partial _t u = - A u + {:} B (u, u) {:} + \sqrt{2 A} \xi . \end{aligned}$$

where $A:V\rightarrow V^*$ is a symmetric dissipative operator, $B: V\otimes _s H\rightarrow V^*$ (the tensor product is defined later) is a bilinear operator with certain symmetry and antisymmetry properties,  : *B* :  represents renormalization, and $\xi$ is a space-time white noise on *H*. The candidate invariant measure for this equation is the law of the white noise on *H*, which in infinite dimensions is supported only on a larger Hilbert space than *H* [11]. Therefore, some care is needed to interpret the (renormalized) nonlinearity  : *B* : . We show that *B* can be interpreted as a distribution on $L^2(\mu )$ and we use regularization by noise techniques, in particular infinite-dimensional semigroups/Kolmogorov equations, to prove weak well-posedness of energy solutions to (1). This is in the spirit of works by Da Prato et al on infinite-dimensional regularization by noise [6–8]. A crucial ingredient is the energy estimate of energy solutions, see Definition 2.18, which serves as a selection principle guaranteeing the uniqueness (see also the discussion on the selection principle in [23]).

One of our main contributions is the introduction of Gelfand triples in this context, eliminating the need for Fourier techniques^1^ while extending the theory to accommodate a wide range of domains, boundary conditions and operator types. Our formulation is motivated by potential applications to the scaling limits of particle systems, inspired in part by recent work on SPDEs with regional fractional Laplacians as scaling limits by Cardoso and Gonçalves [2]. On the technical side, we mostly avoid the use of chaos expansions that are prominent in [24], and instead rely on an integration by parts rule for the reference measure. This choice not only enhances robustness but also simplifies the necessary estimates.

### Statement of the main results

We consider a Gelfand triple consisting of densely embedded, separable Hilbert spaces $V \subset H \subset V^{*}$ and a dense subspace $S \subset V$ that plays the role of smooth functions. For background and further discussion of Gelfand triples, see [29, Ch. 4.1]. We assume that $\Vert \cdot \Vert _V \geqslant \Vert \cdot \Vert _H$ and we define the (semi-)norm

$$ \Vert h \Vert _{\dot{V}}^2 :=\Vert h \Vert _V^2 - \Vert h \Vert _H^2 \ni [0, \Vert h \Vert _V^2], \qquad h \in V, $$

which represents a “homogeneous norm”. The dual norm is canonically defined as

$$ \Vert h \Vert _{\dot{V}^{*}} :=\sup \{ \langle h, g \rangle _{V^{*}, V} : g \in V, \Vert g \Vert _{\dot{V}} = 1 \}, \qquad h \in V^{*} . $$

Although it is possible to complete *V* and $V^{*}$ under the (semi-)norms $\Vert \cdot \Vert _{\dot{V}}$ and $\Vert \cdot \Vert _{\dot{V}^{*}}$, respectively, to obtain Hilbert spaces $\dot{V} \supset V$ and $\dot{V}^{*} \subset V^{*}$, this will not be necessary for our purposes and we will rely only on the definitions of the norms.

#### Assumption

**(A).** Let $A: V \rightarrow V^{*}$ be a bounded linear operator satisfying for all $f, g \in S$

2 $$\begin{aligned} \langle A f, g \rangle _{V^{*}, V} = \langle f, A g \rangle _{V, V^{*}} . \end{aligned}$$

Additionally, we assume that $A f \in H$ for all $f \in S$, meaning that smooth functions are mapped to *H*, and we assume that there exist $c, C > 0$ such that for all $f, g \in S$

3 $$\begin{aligned} | \langle A f, g \rangle _{V^{*}, V} | \leqslant C \Vert f \Vert _{\dot{V}} \Vert g \Vert _{\dot{V}}, \end{aligned}$$

and

4 $$\begin{aligned} \langle A f, f \rangle _{V^{*}, V} \geqslant c \Vert f \Vert _{\dot{V}}^2 . \end{aligned}$$

By approximation, these properties extend to $f, g \in V$.

#### Assumption

**(B).** Let $B : V \times V \rightarrow V^{*}$ be a continuous symmetric bilinear operator with

5 $$\begin{aligned} | \langle B (f, g), h \rangle _{V^{*}, V} | \lesssim (\Vert f \Vert _V \Vert g \Vert _H + \Vert f \Vert _H \Vert g \Vert _V) \Vert h \Vert _{\dot{V}}, \qquad f, g, h \in S, \end{aligned}$$

and such that

6 $$\begin{aligned} \langle B (f, f), f \rangle _{V^{*}, V} = 0, \qquad f \in S. \end{aligned}$$

Again, these properties extend to $f, g, h \in V$ by approximation. We also assume that *B* has a continuous extension to $V \otimes _s H$, see Definition 2.13, satisfying

7 $$\begin{aligned} \Vert B (\varphi )\Vert _{\dot{V}^{*}} \lesssim \Vert \varphi \Vert _{V \otimes _s H}. \end{aligned}$$

Moreover, we assume that there exist $\alpha >0$ and sequences $(\rho _N)_{N \in {\mathbb {N}}} \subset L(H,V)$ and $\varepsilon _N \rightarrow 0$, such that for all $f \in S$ and $\varphi \in S \otimes _s S$,

8 $$\begin{aligned}&\sup _N \left\{ \Vert \rho _N \Vert _{L (H, H)} + \Vert \rho _N \Vert _{L (\dot{V}, \dot{V})} + \varepsilon _N^\alpha \Vert \rho _N\Vert _{L(H,V)}\right\} < \infty , \end{aligned}$$

9 $$\begin{aligned}&\Vert (\rho _N - \text {Id})f \Vert _V + \Vert \langle B \circ \rho _N^{\otimes 2}, f \rangle _{V^{*}, V} \!-\! \langle B, f \rangle _{V^{*}, V} \Vert _{L (V \otimes _s H, \mathbb {R})} \!\leqslant \! C_1 (f) \varepsilon _N, \end{aligned}$$

10 $$\begin{aligned}&{\Vert \langle B (\varphi ), (\rho _N\!-\!\text {Id})\cdot \rangle _{V^{*}, V} \Vert _{L (\dot{V}, \mathbb {R})}+ } \Vert \langle B (\rho _N^{\otimes 2}\varphi ), \cdot \rangle _{V^{*}, V}\! -\! \langle B (\varphi ), \cdot \rangle _{V^{*}, V} \Vert _{L (\dot{V}, \mathbb {R})} \!\leqslant \! C_2 (\varphi ) \varepsilon _N, \end{aligned}$$

where $C_1 : S \rightarrow [0, \infty )$ and $C_2 : S \otimes _s S \rightarrow [0, \infty )$ are such that $\mathbb {E} [C_1 (D F)^2 + C_2 (D^2 F)^2] < \infty$ for all cylinder functions $F \in \mathcal {C}$ (to be defined below, and the expectation is with respect to the white noise on *H*).

#### Remark 1.1

The assumptions on $A$ and $B$ are natural, while the assumption on $(\rho _N)$ is more technical. It may be possible to avoid this assumption by finding different arguments. However, in concrete examples, there often exists a natural family of approximations $(\rho _N)$ for which the estimates in Assumption 1 hold. For instance, in the derivation of energy solutions from particle systems, $\rho _N$ is frequently defined by integrating against a rescaled indicator function over a small set, see [2, 18, 25].

Our aim is to prove uniqueness and, under additional assumptions, existence of energy solutions to the equation

11 $$\begin{aligned} \partial _t u = - A u + {:}B (u, u){:} + \sqrt{2 A} \xi , \end{aligned}$$

where  : *B*(*u*, *u*) :  is a suitable renormalization of the bilinearity, and $\xi$ is a space-time white noise over *H*, i.e. a centered Gaussian process indexed by $L^2 (\mathbb {R}_+; H)$ with covariance

$$ \mathbb {E} [\xi (g) \xi (h)] = \int _0^{\infty } \langle g (t), h (t) \rangle \textrm{d}t. $$

We analyze the equation under the candidate invariant measure $\mu$, the law of the white noise on *H*. Under $\mu$ the process $(\eta (h))_{h \in H}$ is centered Gaussian with covariance

$$\begin{aligned} \int \eta (h) \eta (g) \mu (\textrm{d}\eta ) = \langle h, g \rangle _H . \end{aligned}$$

Energy solutions are weak (both analytically and probabilistically) solutions that respect the natural probabilistic structure of (11); see Definition 2.18 for a precise formulation.

#### Remark 1.2

The measure $\mu$ makes sense as a Gaussian probability measure on any Hilbert space *W* such that there is a dense Hilbert-Schmidt embedding $\iota : H \hookrightarrow W$ since then we can consider the unique Gaussian measure with trace-class operator $\iota \iota ^{*}$ on *W*. Such an embedding always exists and the specific choice of *W* does not matter since we deal with a *W*-valued random variable $\eta$ for which the law of the process $(\langle \eta , \iota (h) \rangle _W)_{h \in H}$ is uniquely determined, see e.g. [11, Sec. 4.1.2].

#### Theorem 1.3

(Existence of energy solutions, Theorem 3.5 below) Suppose that *S* is a nuclear Fréchet space and that there exists a complete orthonormal system $(e_n)_{n \in \mathbb {N}} \subset V$ of *H* consisting of eigenvectors for *A* with eigenvalues $(\lambda _n)_{n \in \mathbb {N}}$. Let $\nu \ll \mu$ be such that $\frac{\textrm{d}\nu }{\textrm{d}\mu } \in L^2(\mu )$. Then under Assumptions 1 and 1, there exists an energy solution *u* to (11) with $u_0 \sim \nu$.

#### Theorem 1.4

(Uniqueness of energy solutions, Theorem 3.7 and Remark 3.8 below) Suppose Assumptions 1 and 1 are satisfied. Let $\nu \ll \mu$ with $\frac{\textrm{d}\nu }{\textrm{d}\mu } \in L^2 (\mu )$ and let $(u_t)_{t \geqslant 0}$ be an energy solution to (11) with $u_0 \sim \nu$. Then the law of *u* is uniquely determined by its initial condition, *u* is a Markov process and it has $\mu$ as an invariant measure. If $\Vert v\Vert _{\dot{V}}=0$ only for $v=0 \in V$, then *u* is ergodic.

#### Remark 1.5

(Normalization of coefficients) We chose very specific coefficients for convenience, but by a small adaptation we can treat the equation

$$ \partial _t u = - \nu A u + \lambda {:} B (u, u) {:} + \sqrt{\sigma ^2 2 A} \xi , $$

for any $\nu , \sigma ^2 > 0$ and $\lambda \in \mathbb {R}$. In that case we simply redefine $\tilde{A} :=\nu A$ and $\tilde{B} = \lambda B$ and $\tilde{\xi } = \sqrt{\frac{\sigma ^2}{\nu }} \xi$ to obtain an equation with normalized coefficients $\partial _t u = - \tilde{A} u + : \tilde{B} (u, u) : + \sqrt{2 \tilde{A}} \tilde{\xi }$. The noise $\tilde{\xi }$ is no longer a space-time white noise over *H*, but over $\tilde{H} = H$ with rescaled norm $\Vert h \Vert _{\tilde{H}}^2 = \frac{\sigma ^2}{\nu } \Vert h \Vert _H^2$ and $\Vert v \Vert _{\tilde{V}}^2 = \frac{\sigma ^2}{\nu } \Vert v \Vert _V^2$. The operators $\tilde{A}$ and $\tilde{B}$ satisfy similar estimates with respect to $\tilde{V}$ as *A* and *B* do with respect to *V*. Therefore, the same existence and uniqueness results hold in that case.

#### Remark 1.6

All results of this paper, in particular the uniqueness statements, extend to the equation obtained by adding a linear term *Cu* to the drift in (11), where $C:\dot{V}\rightarrow V^*$ is bounded, $C(S)\subset H$, and *C* is antisymmetric on *S*, i.e.

$$ \langle Cf,g\rangle = -\langle Cg,f\rangle , \qquad f,g\in S $$

(equivalently $\langle Cf,f\rangle =0$ for all $f\in S$). In the weak formulation (47) this corresponds to the additional term

$$ \mathrm d u_t(f)=\cdots - u_t(Cf)\,\mathrm dt , \qquad f\in S. $$

On cylinder functions $F\in {\mathcal {C}}$, the corresponding generator in (45) becomes

$$ \mathcal {L} = \mathcal {L}_0 + \mathcal {G} + \mathcal {A}_C, \qquad \mathcal {A}_C F := \delta (CDF). $$

The operator ${\mathcal {A}}_C$ is antisymmetric on ${\mathcal {C}}$ and extends to a bounded map $\mathcal {H}^1_\alpha \rightarrow \mathcal {H}^{-1}_\alpha$ for all $\alpha \in {\mathbb {R}}$. Hence, it is a bounded antisymmetric perturbation of $\mathcal {L}_0$ and can be handled by a simpler variant of the arguments used for $\mathcal {G}$.

#### Remark 1.7

In the existence result Theorem 1.3, the condition that *A* has an eigenbasis is technical and can likely be omitted. A possible workaround would be to approximate a self-adjoint *A* satisfying Assumption (A) by the spectral discretizations

$$ A^N := \sum _{i=1}^\infty \frac{i}{N}\, {\textbf{1}}_{\bigl (\frac{i-1}{N},\frac{i}{N}\bigr ]}(A), $$

where ${\textbf{1}}_I(A)$ denotes a spectral projection (functional calculus). Then $A^N$ is self-adjoint and $\sigma (A^N)\subset \frac{1}{N}{\mathbb {N}}$, hence $A^N$ is diagonalizable and Theorem 3.5 yields an energy solution $u^N$ for the equation with *A* replaced by $A^N$. To pass to a limit $N\rightarrow \infty$ one would need uniformity of the estimates presented below, in particular the tightness bounds of Lemma 3.4. This is in the spirit of Gubinelli–Turra [26], who approximate the Laplacian on ${\mathbb {R}}^2$ by Laplacians on tori of increasing size.

### Structure of the paper

The paper is organized as follows. In Section 2 we provide a functional analytic setup for our problem. Section 2.1 is dedicated to deriving expressions for the operators appearing in the Itô formula for energy solutions, $\mathcal {L}_0$ and $\mathcal {G}$, corresponding to the linear part of the dynamics and the bilinear operator *B* as in (11) respectively. In Section 2.2 we derive bounds for the operators $\mathcal {L}_0$ and $\mathcal {G}$ on Sobolev type spaces based on $L^2(\mu )$. Finally in Section 2.3 we define energy solutions in Definition 2.18 and give an alternative characterization in Theorem 2.20 that is more applicable in the particle systems setting. Section 3 is dedicated to the construction of energy solutions and the corresponding semigroups. In Section 3.1 we construct the semigroup and resolvent. Energy solutions are constructed in Section 3.2. Finally, in Section 3.3 we prove the uniqueness of energy solutions in Theorem 3.7, by establishing duality between semigroup and energy solution.

The final section is devoted to examples. For simplicity we focus on the one-dimensional Burgers equation with various linear operators and boundary conditions. In Section 4 we provide a detailed analysis for the (multi-component) stochastic Burgers equation on (0, 1) or $(0, \infty )$ with homogeneous Dirichlet boundary conditions, possibly with a divergence form differential operator instead of the usual Laplacian. We also discuss homogeneous Neumann boundary conditions, which we can handle in a hyperviscous setting with $A = (1 - \Delta )^{\theta }$ for $\theta > 1$. In the Dirichlet case we can also choose $- A$ as the regional fractional Laplacian of order $\gamma \in \left[ \frac{3}{2}, 2 \right)$, which is motivated by recent results on fluctuations in interacting particle systems by [2].

## Functional analytic setup

### Derivation of the generator

In this section we derive useful expressions for the operators appearing in the Itô formula for energy solutions. We work on the probability space $\Omega =W$ with *W* as in Remark 1.2, where we consider the coordinate map $\eta :\Omega \rightarrow W$, $\eta (\omega ) = \omega$, and $\eta (h):=\langle \eta ,\mathcal \iota (h)\rangle _W$, $h \in H$ and ${\mathcal {F}} = \sigma (\eta (h):h \in H)$. We write $\mu$ for the measure on $(\Omega , {\mathcal {F}})$ under which $\eta$ is a white noise on *H*, see Remark 1.2.

#### Definition 2.1

(Cylinder functions) Any *cylinder function* is of the form $F (\eta ) = \Phi (\eta (f))$, where $f \in S^m$ and $\Phi : \mathbb {R}^m \rightarrow \mathbb {R}$ is a polynomial. We write $\mathcal {C}$ for the set of all cylinder functions.

For a cylinder function $F (\eta ) = \Phi (\eta (f))$ we define the operator

12 $$\begin{aligned} \mathcal {L}_0 F (\eta ) :=\sum _{i = 1}^m \partial _i \Phi (\eta (f)) \eta (- A f_i) + \frac{1}{2} \sum _{i, j = 1}^m \partial _{i j}^2 \Phi (\eta (f)) 2 \langle A f_i, f_j \rangle _H. \end{aligned}$$

Formally, this is the differential operator that appears when we apply Itô’s formula to the (weak formulation of the) linear part of the dynamics (11) (i.e. with $B = 0$). To proceed, we need the following well known observation (see [32] for the definitions of *D*, $\delta$):

#### Lemma 2.2

(Multiplication formula for Skorokhod integral) Let $\delta$ be the Skorokhod integral and let *D* be the Malliavin derivative, both with respect to the white noise on *H*. Let $F \in \mathcal {C}$ and let $\varphi \in \operatorname {dom} (\delta )$. Then

13 $$\begin{aligned} F \delta (\varphi ) = \delta (F \varphi ) + \langle D F, \varphi \rangle _H . \end{aligned}$$

In particular, we have for any $h \in H$

14 $$\begin{aligned} F (\eta ) \eta (h) = \delta (F h) (\eta ) + \langle D F (\eta ), h \rangle _H = \delta (F h) (\eta ) + D_h F (\eta ) . \end{aligned}$$

#### Proof

Let *G* be another cylinder function. Then

$$\begin{aligned} \mathbb {E} [(F \delta (\varphi ) - \langle D F, \varphi \rangle _H) G]&= \mathbb {E} [\delta (\varphi ) F G] -\mathbb {E} [\langle D F, \varphi \rangle _H G]\\&= \mathbb {E} [\langle \varphi G, D F \rangle _H] +\mathbb {E} [\langle \varphi F, D G \rangle _H] -\mathbb {E} [\langle D F, \varphi \rangle _H G]\\&= \mathbb {E} [\delta (\varphi F) G] . \end{aligned}$$

Since this is true for all cylinder functions *G*, the claimed identity follows. $\square$

This gives a nice expression for $\mathcal {L}_0 F$:

#### Corollary 2.3

For any cylinder function $F \in \mathcal {C}$, we have

15 $$\begin{aligned} \mathcal {L}_0 F = \delta (- A D F) . \end{aligned}$$

#### Proof

In (12) we can write $\sum _{i, j = 1}^m\! \partial _{i j}^2 \Phi (\eta (f)) \langle A f_i, f_j \rangle _H\! \!=\! \!\sum _{i = 1}^m \!\langle D \partial _i \Phi (\eta (f)), A f_i \rangle _H$ and therefore

$$\begin{aligned} \mathcal {L}_0 F (\eta )&= \sum _{i = 1}^m \partial _i \Phi (\eta (f)) \eta (- A f_i) + \sum _{i = 1}^m \langle D \partial _i \Phi (\eta (f)), A f_i \rangle _H\\&= \delta \left( \sum _{i = 1}^m \partial _i \Phi (\eta (f)) (- A f_i) \right) (\eta ) = \delta (- A D F) (\eta ) . \end{aligned}$$

$$\square $$

Our next goal is to derive an expression for the generator $\mathcal {G}$ corresponding to the nonlinearity *B*. Here we have to use a suitable approximation, and even the definition of the approximation takes some work.

#### Lemma 2.4

Let $\rho : H \rightarrow V$ be a bounded operator and let $v \in H$. Let $(e_k)_k$ be an orthonormal basis of *H*. Then the following limits exist in $L^2 (\mu )$ and do not depend on the specific orthonormal basis:

16 $$\begin{aligned} \langle {:} B^{\rho } (\eta , \eta ) {:}, v \rangle _{V^{*}, V} :=\lim _{N \rightarrow \infty } \sum _{k, \ell \leqslant N} (\eta (e_k) \eta (e_{\ell }) - \delta _{k, \ell }) \langle B (\rho e_k, \rho e_{\ell }), \rho v \rangle _{V^{*}, V} \end{aligned}$$

and, if additionally $v \in V$, the same is true for

17 $$\begin{aligned} \langle {:} \tilde{B}^{\rho } (\eta , \eta ) {:}, v \rangle _{V^{*}, V} :=\lim _{N \rightarrow \infty } \sum _{k, \ell \leqslant N} (\eta (e_k) \eta (e_{\ell }) - \delta _{k, \ell }) \langle B (\rho e_k, \rho e_{\ell }), v \rangle _{V^{*}, V}. \end{aligned}$$

#### Proof

The term $\sum _{k, \ell \leqslant N} (\eta (e_k) \eta (e_{\ell }) - \delta _{k, \ell }) \langle B (\rho e_k, \rho e_{\ell }), \rho v \rangle _{V^{*}, V}$ is in the second homogeneous chaos, and $\eta (e_k) \eta (e_{\ell }) - \delta _{k, \ell } = \delta (e_k \delta (e_{\ell }))$ by Lemma 2.2. Consider a cylinder function *G* that is also in the second homogeneous chaos (this is sufficient for the expectation below, because any other chaos would give an orthogonal contribution to our sum). Integrating $\delta$ by parts twice, we obtain with $\Pi _N h = \sum _{k \leqslant N} \langle h, e_k \rangle _{V,V^{*}} e_k$

$$\begin{aligned}&\mathbb {E} \left[ \left( \sum _{k, \ell \leqslant N} (\eta (e_k) \eta (e_{\ell }) - \delta _{k, \ell }) \langle B (\rho e_k, \rho e_{\ell }), \rho v \rangle _{V^{*}, V} \right) G \right] \\&= \sum _{k, \ell \leqslant N} \mathbb {E} [D_{e_k} D_{e_{\ell }} G \langle B (\rho e_k, \rho e_{\ell }), \rho v \rangle _{V^{*}, V}]\\&=\mathbb {E} [\langle B (\rho ^{\otimes 2} \Pi _N^{\otimes 2} D^2 G), \rho v \rangle _{V^{*}, V}]\\&\lesssim \Vert \rho \Vert _{L (H, V)} \Vert \rho \Vert _{L (H, H)} \mathbb {E} [\Vert \Pi ^{\otimes 2}_N D^2 G \Vert _{H \otimes _s H}^2] \Vert \rho \Vert _{L (\dot{V}, \dot{V})} \Vert v \Vert _{\dot{V}}\\&\lesssim \Vert \rho \Vert _{L (H, V)} \Vert \rho \Vert _{L (H, H)} \Vert \rho \Vert _{L (\dot{V}, \dot{V})} \mathbb {E} [\Vert D^2 G \Vert _{H \otimes _s H}^2] \Vert v \Vert _{\dot{V}}\\&\simeq \Vert \rho \Vert _{L (H, V)} \Vert \rho \Vert _{L (H, H)} \Vert \rho \Vert _{L (\dot{V}, \dot{V})} {\Vert G \Vert _{L^2 (\mu )}} \Vert v \Vert _{\dot{V}}, \end{aligned}$$

where in the last step we used that the Malliavin derivative is a bounded operator when acting on a fixed chaos. This shows that $\bigg ( \sum _{k, \ell \leqslant N} (\eta (e_k) \eta (e_{\ell }) - \delta _{k, \ell }) \langle B (\rho e_k, \rho e_{\ell }), \rho v \rangle _{V^{*}, V} \bigg )_N$ is uniformly bounded in $L^2 (\mu )$, and a similar argument using that $\Pi _N^{\otimes 2}$ approximates the identity in $H \otimes _s H$ shows that it is a Cauchy sequence and its limit does not depend on the specific orthonormal basis used for the construction. The same argument works also for ${:} \tilde{B}^{\rho } (\eta , \eta ) {:}$ in place of ${:} B^{\rho } (\eta , \eta ){ :}$. $\square$

#### Remark 2.5

For later reference note that we can extract the following bounds from the proof:

18 $$\begin{aligned} \Vert \langle {:} B^{\rho } (\eta , \eta ) {:}, v \rangle _{V^{*}, V}\Vert _{L^2(\mu )}&\lesssim \Vert \rho \Vert _{L (H, V)} \Vert \rho \Vert _{L (H, H)} \Vert \rho \Vert _{L (\dot{V}, \dot{V})} \Vert v \Vert _{\dot{V}}, \end{aligned}$$

19 $$\begin{aligned} \Vert \langle {:} \tilde{B}^{\rho } (\eta , \eta ) {:}, v \rangle _{V^{*}, V}\Vert _{L^2(\mu )}&\lesssim \Vert \rho \Vert _{L (H, V)} \Vert \rho \Vert _{L (H, H)} \Vert v \Vert _{\dot{V}}. \end{aligned}$$

#### Lemma 2.6

Let $\rho : H \rightarrow V$ be a bounded operator. We define for cylinder functions $F = \Phi (\eta (f)) \in \mathcal {C}$ with $\langle {:} B^{\rho } (\eta , \eta ) {:}, f_i \rangle _{V^{*}, V}$ as in (16)

20 $$\begin{aligned} \mathcal {G}^{\rho } F (\eta ) :=\sum _{i = 1}^m \partial _i \Phi (\eta (f)) \langle {:} B^{\rho } (\eta , \eta ) {:}, f_i \rangle _{V^{*}, V} . \end{aligned}$$

Then we can decompose

21 $$\begin{aligned} \mathcal {G}^{\rho } F =\mathcal {G}^{\rho }_+ +\mathcal {G}^{\rho }_-, \end{aligned}$$

where with any orthonormal basis $(e_k)_{k \in \mathbb {N}}$ of *H*,

22 $$\begin{aligned} \mathcal {G}_+^{\rho } F&= \sum _{k, \ell \in \mathbb {N}} \delta (e_{\ell } \delta (e_k \langle B (\rho e_k, \rho e_{\ell }), \rho D F \rangle _{V^{*}, V})), \end{aligned}$$

23 $$\begin{aligned} \mathcal {G}_-^{\rho } F&= 2 \sum _{k, \ell \in \mathbb {N}} \delta (e_k \langle B (\rho e_k, \rho e_{\ell }), \rho D D_{e_{\ell }} F \rangle _{V^{*}, V}) . \end{aligned}$$

#### Proof

By definition,

24 $$\begin{aligned} \mathcal {G}^{\rho } F (\eta ) = \sum _{k, \ell \in \mathbb {N}} \sum _{i = 1}^m \partial _i \Phi (\eta (f)) \langle B (\rho e_k, \rho e_{\ell }), \rho f_i \rangle _{V^{*}, V} (\eta (e_k) \eta (e_{\ell }) - \delta _{k, \ell }) \end{aligned}$$

By the multiplication rule for Skorokhod integrals in Lemma 2.2$\eta (e_k) \eta (e_{\ell }) - \delta _{k, \ell } = \delta (\delta (e_k) e_{\ell }) (\eta )$. Using once more multiplication rule in (13) in the form $G \delta (\varphi ) = \delta (G \varphi ) + \langle D G, \varphi \rangle _H = \delta (G \varphi ) + D_{\varphi } G$, we can thus rewrite $\mathcal {G}^{\rho } F$ as

25 $$\begin{aligned} \mathcal {G}^{\rho } F&= \sum _{k, \ell \in \mathbb {N}} \delta (\langle B (\rho e_k, \rho e_{\ell }), \rho D F \rangle _{V^{*}, V} \delta (e_k) e_{\ell })\nonumber \\&\quad + \sum _{k, \ell \in \mathbb {N}} \langle D (\langle B (\rho e_k, \rho e_{\ell }), \rho D F \rangle _{V^{*}, V}), e_{\ell } \delta (e_k) \rangle _H \nonumber \\&= \sum _{k, \ell } \delta (e_{\ell } \langle B (\rho e_k, \rho e_{\ell }), \rho D F \rangle _{V^{*}, V} \delta (e_k)) + \sum _{k, \ell } \langle B (\rho e_k, \rho e_{\ell }), \rho D D_{e_{\ell }} F \rangle _{V^{*}, V} \delta (e_k) \nonumber \\&= \sum _{k, \ell } \delta (e_{\ell } \delta (e_k \langle B (\rho e_k, \rho e_{\ell }), \rho D F \rangle _{V^{*}, V})) + \sum _{k, \ell } \delta (e_{\ell } \langle B (\rho e_k, \rho e_{\ell }), \rho D D_{e_k} F \rangle _{V^{*}, V})\nonumber \\&\qquad + \sum _{k, \ell } \delta (e_k \langle B (\rho e_k, \rho e_{\ell }), \rho D D_{e_{\ell }} F \rangle _{V^{*}, V}) + \sum _{k, \ell } \langle B (\rho e_k, \rho e_{\ell }), \rho D D_{e_k} D_{e_{\ell }} F \rangle _{V^{*}, V} . \end{aligned}$$

Expanding the third order Malliavin derivative in the last term on the right hand side, we obtain

$$\begin{aligned} \sum _{k, \ell } \langle B (\rho e_k, \rho e_{\ell }), \rho D D_{e_k} D_{e_{\ell }} F \rangle _{V^{*}, V}&= \sum _{i_1, i_2, i_3 = 1}^m \partial _{i_1 i_2 i_3}^3 \Phi (\eta (f)) \langle B (\rho f_{i_2}, \rho f_{i_3}), \rho f_{i_1} \rangle _{V^{*}, V} . \end{aligned}$$

To proceed, we need Lemma A.1 from the appendix, which shows that

$$\begin{aligned} \langle B (\rho f_{i_2}, \rho f_{i_3}), \rho f_{i_1} \rangle _{V^{*}, V} + \langle B (\rho f_{i_1}, \rho f_{i_2}), \rho f_{i_3} \rangle _{V^{*}, V} + \langle B (\rho f_{i_3}, \rho f_{i_1}), \rho f_{i_2} \rangle _{V^{*}, V} = 0, \end{aligned}$$

and consequently, using the symmetry of $\partial _{i_1 i_2 i_3}^3 \Phi (\eta (f))$ in $i_1, i_2, i_3$,

$$\begin{aligned} \sum _{i_1, i_2, i_3 = 1}^m \partial _{i_1 i_2 i_3}^3 \Phi (\eta (f)) \langle B (\rho f_{i_2}, \rho f_{i_3}), \rho f_{i_1} \rangle _{V^{*}, V} = 0. \end{aligned}$$

We remain with

$$\begin{aligned} \mathcal {G}^{\rho } F&= \sum _{k, \ell } \delta (e_{\ell } \delta (\langle B (\rho e_k, \rho e_{\ell }), \rho D F \rangle _{V^{*}, V} e_k))\\  &\quad + 2 \sum _{k, \ell } \delta (e_k (\langle B (\rho e_k, \rho e_{\ell }), \rho D D_{e_{\ell }} F \rangle _{V^{*}, V}))\\&= \mathcal {G}^{\rho }_+ F +\mathcal {G}^{\rho }_- F, \end{aligned}$$

where we used the symmetry of *B* in the first line. $\square$

The operators $\mathcal {G}_+^{\rho }$ and $\mathcal {G}_-^{\rho }$ are antisymmetric with respect to each other:

#### Lemma 2.7

If *F* and *G* are cylinder functions, then

26 $$\begin{aligned} \mathbb {E} [(\mathcal {G}_+^{\rho } F) G] = -\mathbb {E} [F\mathcal {G}^{\rho }_- G] . \end{aligned}$$

#### Proof

We use several times that $\delta$ and *D* are adjoint operators, and in the fifth line we use Lemma A.1 from the appendix together with the symmetry of $D_{e_k} D_{e_{\ell }} G$ in $k, \ell$

27 $$\begin{aligned} \mathbb {E} [(\mathcal {G}_+^{\rho } F) G]&= \sum _{k, \ell } \mathbb {E} [\delta (e_{\ell } \delta (e_k \langle B (\rho e_k, \rho e_{\ell }), \rho D F \rangle _{V^{*}, V})) G] \nonumber \\&= \sum _{k, \ell } \mathbb {E} [\delta (e_k \langle B (\rho e_k, \rho e_{\ell }), \rho D F \rangle _{V^{*}, V}) D_{e_{\ell }} G] \nonumber \\&= \sum _{k, \ell } \mathbb {E} [\langle B (\rho e_k, \rho e_{\ell }), \rho D F \rangle _{V^{*}, V} D_{e_k} D_{e_{\ell }} G] \\&= \sum _{k, \ell , m} \mathbb {E} [\langle B (\rho e_k, \rho e_{\ell }), \rho e_m \rangle _{V^{*}, V} D_{e_m} F D_{e_k} D_{e_{\ell }} G] \nonumber \\&= - 2 \sum _{k, \ell , m} \mathbb {E} [\langle B (\rho e_k, \rho e_m), \rho e_{\ell } \rangle _{V^{*}, V} D_{e_m} F D_{e_k} D_{e_{\ell }} G] \nonumber \\&= - 2 \sum _{k, m} \mathbb {E} [\langle B (\rho e_k, \rho e_m), \rho D D_{e_k} G \rangle _{V^{*}, V} D_{e_m} F] \nonumber \\&= - 2 \sum _{k, m \leqslant N} \mathbb {E} [\delta (e_m \langle B (\rho e_k, \rho e_m), \rho D D_{e_k} G \rangle _{V^{*}, V}) F] \nonumber \\&= -\mathbb {E} [F\mathcal {G}_- G] . \nonumber \end{aligned}$$

$$\square $$

#### Remark 2.8

For later reference, we note that equation (27) can be written as

28 $$\begin{aligned} \mathbb {E} [(\mathcal {G}_+^{\rho } F) G] = \mathbb {E} [\langle B (\rho ^{\otimes 2} D^2G), \rho D F \rangle _{V^{*}, V}]. \end{aligned}$$

To derive estimates for $\mathcal {G^\rho }$ and $\mathcal {L}_0$, we need to introduce suitable function spaces.

### Function spaces and operator bounds

Here we define Sobolev type spaces over $L^2 (\mu )$, which can be interpreted as tensorizations of $V, H, V^{*}$ and also of the homogeneous spaces $\dot{V}, \dot{V}^{*}$. We start with the definition of an auxiliary operator.

#### Definition 2.9

(Number operator/Ornstein-Uhlenbeck operator) The *number operator*, also called *Ornstein-Uhlenbeck operator*, is defined as

29 $$\begin{aligned} \mathcal {N} :=\delta D, \qquad \operatorname {dom} (\mathcal {N}) :=\{ F \in \operatorname {dom} (D) : D F \in \operatorname {dom} (\delta ) \} . \end{aligned}$$

$\mathcal {N}$ is a self-adjoint, positive semi-definite linear operator, see [32, p. 59], and therefore we can define the powers $(1 +\mathcal {N})^{\alpha }$ using functional calculus. We have $\mathcal {C} \subset \operatorname {dom} ((1 +\mathcal {N})^{\alpha })$ for any $\alpha \in \mathbb {R}$, and in fact $(1 +\mathcal {N})^{\alpha } \mathcal {C} \subset \mathcal {C}$. This can be shown using the chaos expansion under the Gaussian measure $\mu$, since $\mathcal {N}$ acts as multiplication with *n* on the *n*-th homogeneous chaos, and any element of $\mathcal {C}$ has a finite chaos expansion.

#### Lemma 2.10

The operators $\mathcal {L}_0$ and $\mathcal {N}$ commute on cylinder functions:

30 $$\begin{aligned} {[}\mathcal {L}_0, \mathcal {N}] F = \mathcal {L}_0 \mathcal {N} F - \mathcal {N} \mathcal {L}_0 F = 0, \end{aligned}$$

for all $$F \in \mathcal {C}$$.

#### Proof

By Lemma A.2, we have

$$ \mathcal {L}_0 \mathcal {N} F = - \delta (A D \mathcal {N} F) = - \delta (A (\mathcal {N} + 1) D \mathcal   F) = - \delta ((\mathcal {N} + 1) A D \mathcal   F) = - \mathcal {N} \delta (A D \mathcal   F) . $$

$$\square $$

#### Definition 2.11

For any cylinder function $F \in \mathcal {C}$ and any $\alpha \in \mathbb {R}$ we define

31 $$\begin{aligned} \Vert F \Vert _{\dot{\mathcal {H}}^1_{\alpha }}^2&:=\mathbb {E} [\Vert D (1 +\mathcal {N})^{\alpha } F \Vert _{\dot{V}}^2], \end{aligned}$$

32 $$\begin{aligned} \Vert F \Vert _{\mathcal {H}^1_{\alpha }}^2&:=\Vert (1 +\mathcal {N})^{\alpha } F \Vert _{L^2 (\mu )}^2 + \Vert F \Vert _{\dot{\mathcal {H}}^1_{\alpha }}^2, \end{aligned}$$

33 $$\begin{aligned} \Vert F \Vert _{\dot{\mathcal {H}}^{- 1}_{- \alpha }}^2&:=\sup \{ \mathbb {E} [F G] : G \in \mathcal {C}, \Vert G \Vert _{\dot{\mathcal {H}}^1_{\alpha }} = 1 \}, \end{aligned}$$

34 $$\begin{aligned} \Vert F \Vert _{\mathcal {H}^{- 1}_{- \alpha }}^2&:=\sup \{ \mathbb {E} [F G] : G \in \mathcal {C}, \Vert G \Vert _{\mathcal {H}^1_{\alpha }} = 1 \} . \end{aligned}$$

We also define the Hilbert spaces $\mathcal {H}^1_{\alpha }$ and $\mathcal {H}^{- 1}_{- \alpha }$ as the completion of $(\mathcal {C}, \Vert \cdot \Vert _{\mathcal {H}^1_{\alpha }})$ and $(\mathcal {C}, \Vert \cdot \Vert _{\mathcal {H}^{- 1}_{- \alpha }})$, respectively.

The homogeneous spaces $\dot{\mathcal {H}}^1_0$ and $\dot{\mathcal {H}}^{-1}_0$ play an important role when studying long time fluctuations in Markov processes, see the monograph [28]. Since here we are interested in solvability and not in the long time behavior (beyond ergodicity), we do not need those spaces and only the norms $\Vert \cdot \Vert _{\dot{\mathcal {H}}^1_{\alpha }}^2$ and $\Vert \cdot \Vert _{\dot{\mathcal {H}}^{-1}_{\alpha }}^2$

#### Lemma 2.12

(Bounds for $\mathcal {L}_0$) The operator ${\mathcal {L}}_0$ satisfies the following upper and lower bounds on cylinder functions:

35 $$\begin{aligned} \Vert (1 -\mathcal {L}_0) F \Vert _{\mathcal {H}^{- 1}_{0}} \lesssim \Vert F \Vert _{\mathcal {H}^1_{0}}, \qquad F \in \mathcal {C}, \end{aligned}$$

and

36 $$\begin{aligned} \langle (1 -\mathcal {L}_0) F, F \rangle _{\mathcal {H}^{- 1}_0, \mathcal {H}^1_0} \gtrsim \Vert F \Vert _{\mathcal {H}^1_0}^2, \qquad F \in {\mathcal {C}}. \end{aligned}$$

Consequently, there is a unique extension of $\mathcal {L}_0$ to a bounded linear operator from $\mathcal {H}^1_{0}$ to $\mathcal {H}^{- 1}_{0}$, which we denote again by $\mathcal {L}_0$, and which satisfies the lower bound (36) for all $F \in {\mathcal {H}}^1_0$.

#### Proof

We use the bound $| \langle A v, w \rangle _{V^{*}, V} | \leqslant C \Vert v \Vert _{\dot{V}} \Vert w \Vert _{\dot{V}}$ together with the Cauchy-Schwarz inequality to obtain the claimed upper bound for $\Vert (1 -\mathcal {L}_0) F \Vert _{\mathcal {H}^{- 1}_0}$ by testing against $G \in {\mathcal {C}}$:

37 $$\begin{aligned} \mathbb {E} [(1 -\mathcal {L}_0) F G]&= \mathbb {E} [F G] +\mathbb {E} [\delta (A D F) G] \nonumber \\&= \mathbb {E} [F G] +\mathbb {E} [\langle A D F, D G \rangle _H] \nonumber \\&\leqslant \mathbb {E} [F^2]^{1 / 2} \mathbb {E} [G^2]^{1 / 2} + C\mathbb {E} [\Vert D F \Vert _{\dot{V}}^2]^{1 / 2} \mathbb {E} [\Vert D G \Vert _{\dot{V}}]^{1 / 2} \nonumber \\&\lesssim \Vert F \Vert _{\mathcal {H}^1_0} \Vert G \Vert _{\mathcal {H}^1_0} . \end{aligned}$$

For the lower bound, we similarly estimate

$$\begin{aligned} \mathbb {E} [((1 -\mathcal {L}_0) F) F]&= \mathbb {E} [F^2] +\mathbb {E} [\langle A D F, D F \rangle _H] \nonumber \\&\geqslant \mathbb {E} [F^2] + c\mathbb {E} [\Vert D F \Vert _{\dot{V}}^2] \nonumber \\&\gtrsim \Vert F \Vert _{\mathcal {H}^1_0}^2. \end{aligned}$$

$$\square $$

#### Definition 2.13

($V \otimes _s H$) For $m \in \mathbb {N}$, symmetric $a = (a_{i j})_{i, j = 1, \ldots , m} \in \mathbb {R}^{m \times m}$, and $v_1, \ldots , v_m \in V$ we define

$$ \left\| \sum _{i, j = 1}^m a_{i j} v_i \otimes v_j \right\| _{V \otimes _s H}^2 :=\sum _{i_1, j_1, i_2, j_2 = 1}^m a_{i_1 j_1} a_{i_2 j_2} \langle v_{i_1}, v_{i_2} \rangle _V \langle v_{j_1}, v_{j_2} \rangle _H, $$

and we write $V \otimes _s H \subset H \otimes _s H$ for the completion of $\left\{ \sum _{i, j = 1}^m a_{i j} v_i \otimes v_j \right\}$ with respect to $\Vert \cdot \Vert _{V \otimes _s H}$.

In the following, we will strongly rely on our assumption that the map

38 $$\begin{aligned} V \otimes _s H \ni \sum _{i, j = 1}^m a_{i j} v_i \otimes v_j \mapsto B \left( \sum _{i, j = 1}^m a_{i j} v_i \otimes v_j \right) :=\sum _{i, j = 1}^m a_{i j} B (v_i, v_j) \in \dot{V}^{*} \end{aligned}$$

has a unique continuous extension to all of $V \otimes _s H$, which we still denote by *B*. We first derive uniform bounds for $\mathcal {G}^{\rho }$, then we will discuss the convergence of $(\mathcal {G}^{\rho _N})_N$ if $\rho _N$ is a sequence of regularization operators that converge to the identity.

#### Lemma 2.14

(Uniform bounds for $\mathcal {G}^\rho$) Let $\rho : H \rightarrow V$ be a bounded operator. For $\alpha \in \{ 0, 1 \}$ the following estimates hold:

39 $$\begin{aligned} \Vert \mathcal {G}^{\rho } F \Vert _{\mathcal {H}^{- 1}_{\alpha - 1}} \lesssim (\Vert \rho \Vert _{L (H, H)} + \Vert \rho \Vert _{L (\dot{V}, \dot{V})})^3 \Vert F \Vert _{\mathcal {H}^1_{\alpha }}, \qquad F \in \mathcal {C}, \end{aligned}$$

as well as the commutator estimate

40 $$\begin{aligned} \Vert [\mathcal {N}, \mathcal {G}^\rho ] F \Vert _{\mathcal {H}^{- 1}_{\alpha - 1}} \lesssim (\Vert \rho \Vert _{L (H, H)} + \Vert \rho \Vert _{L (\dot{V}, \dot{V})})^3 \Vert F \Vert _{\mathcal {H}^1_{\alpha }}, \qquad F \in \mathcal {C}. \end{aligned}$$

#### Proof

To bound $\mathcal {G}_+^{\rho } F$ we consider a cylinder function $G \in \mathcal {C}$ and use equation (28) and in the third line we apply Lemma A.3 from the appendix and use that $\Vert \rho \Vert _{L (V, V)} \leqslant \Vert \rho \Vert _{L (H, H)} + \Vert \rho \Vert _{L (\dot{V}, \dot{V})}$

41 $$\begin{aligned} \mathbb {E} [(\mathcal {G}_+^{\rho } F) G]&= \mathbb {E} [\langle B (\rho ^{\otimes 2} D^2G), \rho D F \rangle _{V^{*}, V}] \nonumber \\&\lesssim \mathbb {E} [\Vert \rho ^{\otimes 2} D^2 G \Vert _{V \otimes _s H}^2]^{1 / 2} \mathbb {E} [\Vert \rho D F \Vert _{\dot{V}}^2]^{1 / 2} \nonumber \\&\lesssim (\Vert \rho \Vert _{L (H, H)} + \Vert \rho \Vert _{L (\dot{V}, \dot{V})})^3 (\mathbb {E} [\Vert D^2 G \Vert _{\dot{V} \otimes _s H}^2] \nonumber \\&\quad +\mathbb {E} [\Vert D^2 G \Vert _{H \otimes _s H}^2])^{1 / 2} \mathbb {E} [\Vert D F \Vert _{\dot{V}}^2]^{1 / 2} \nonumber \\&\lesssim (\Vert \rho \Vert _{L (H, H)} + \Vert \rho \Vert _{L (\dot{V}, \dot{V})})^3 (\Vert \boldsymbol{1}_{\mathcal {N} \geqslant 1} (\mathcal {N}- 1)^{1 / 2} G \Vert _{\dot{\mathcal {H}}^1_0}^2 \nonumber \\&\quad + \Vert \mathcal {N}^{1 / 2} (\mathcal {N}- 1)^{1 / 2} G \Vert _{L^2 (\mu )}^2)^{1 / 2} \cdot \Vert F \Vert _{\dot{\mathcal {H}}^1_0} \nonumber \\&\lesssim (\Vert \rho \Vert _{L (H, H)} + \Vert \rho \Vert _{L (\dot{V}, \dot{V})})^3 \Vert F \Vert _{\dot{\mathcal {H}}^1_0} \Vert G \Vert _{\mathcal {H}^1_1}, \end{aligned}$$

using Lemma 2.15 which follows below this proof in the sixth line. As $(1 +\mathcal {N}) \delta = \delta (2 +\mathcal {N})$ and $(3 +\mathcal {N}) D = D (2 +\mathcal {N})$, see Lemma A.2 in the appendix, this also yields

42 $$\begin{aligned} \mathbb {E} [(\mathcal {G}_+^{\rho } F) G]&= \sum _{k, \ell \in \mathbb {N}} \mathbb {E} [(1 +\mathcal {N}) \delta (\delta (\langle B (e_k, e_{\ell }), D F \rangle _{\dot{V}^{*}, \dot{V}} e_k) e_{\ell }) (1 +\mathcal {N})^{- 1} G] \nonumber \\&= \sum _{k, \ell \in \mathbb {N}} \mathbb {E} [\langle B (e_k, e_{\ell }), D (2 +\mathcal {N}) F \rangle _{\dot{V}^{*}, \dot{V}} D_{e_k} D_{e_{\ell }} (1 +\mathcal {N})^{- 1} G] \nonumber \\&= \mathbb {E} [(\mathcal {G}_+ (2 +\mathcal {N}) F) (1 +\mathcal {N})^{- 1} G] \nonumber \\&\lesssim (\Vert \rho \Vert _{L (H, H)} + \Vert \rho \Vert _{L (\dot{V}, \dot{V})})^3 \Vert (2 +\mathcal {N}) F \Vert _{\dot{\mathcal {H}}^1_0}, \Vert (1 +\mathcal {N})^{- 1} G \Vert _{\mathcal {H}^1_1} \nonumber \\&\simeq (\Vert \rho \Vert _{L (H, H)} + \Vert \rho \Vert _{L (\dot{V}, \dot{V})})^3 \Vert F \Vert _{\dot{\mathcal {H}}^1_1} \Vert G \Vert _{\mathcal {H}^1_0} . \end{aligned}$$

We estimate $\mathcal {G}_-^{\rho }$ by duality with $\mathcal {G}^{\rho }_+$

$$\begin{aligned} \mathbb {E} [(\mathcal {G}^{\rho }_- F) G] =\mathbb {E} [F\mathcal {G}^{\rho }_+ G] \lesssim (\Vert \rho \Vert _{L (H, H)} + \Vert \rho \Vert _{L (\dot{V}, \dot{V})})^3 (\Vert F \Vert _{\mathcal {H}^1_1} \Vert G \Vert _{\dot{\mathcal {H}}^1_0}) \wedge (\Vert F \Vert _{\mathcal {H}^1_0} \Vert G \Vert _{\dot{\mathcal {H}}^1_1}) . \end{aligned}$$

The commutator estimate is the same as in [24]: We just showed

$$ \mathcal {N}\mathcal {G}^{\rho }_+ =\mathcal {G}_+^{\rho } (1 +\mathcal {N}) \qquad \Rightarrow \qquad [\mathcal {N}, \mathcal {G}^{\rho }_+] =\mathcal {G}^{\rho }_+, $$

and by duality this also yields $[\mathcal {N}, \mathcal {G}^{\rho }_-] = -\mathcal {G}^{\rho }_-$. $\square$

#### Lemma 2.15

The following identity holds for all $F \in \mathcal {C}$:

$$ \mathbb {E} [\Vert D^2 F \Vert _{\dot{V} \otimes _s H}^2] = \Vert \boldsymbol{1}_{\mathcal {N} \geqslant 1} (\mathcal {N}- 1)^{1 / 2} F \Vert ^2_{\dot{\mathcal {H}}^1_0} . $$

#### Proof

This is the only proof where we explicitly use the chaos expansion under $\mu$, and it could be avoided with some additional work. The chaos expansion is $F = \sum _{n = 0}^{\infty } W_n (\varphi _n)$ with $\varphi _n \in H^{\otimes n}_s$. Then

$$\begin{aligned} \mathbb {E} [\Vert D^2 F \Vert _{\dot{V} \otimes _s H}^2]&= \sum _{k_1, k_2, \ell _1, \ell _2} \mathbb {E} \left[ D_{e_{\ell _1}} D_{e_{k_1}} F D_{e_{\ell _2}} D_{e_{k_2}} F \right] \langle e_{\ell _1}, e_{\ell _2} \rangle _{\dot{V}} \langle e_{k_1}, e_{k_2} \rangle _H\\&= \sum _{k, \ell _1, \ell _2} \sum _{n = 2}^{\infty } n^2 (n - 1)^2 \mathbb {E} [W_{n - 2} (\varphi _n (e_{\ell _1}, e_k, \cdot )) W_{n - 2} (\varphi _n (e_{\ell _2}, e_k, \cdot ))] \langle e_{\ell _1}, e_{\ell _2} \rangle _{\dot{V}}\\&= \sum _{k, \ell _1, \ell _2} \sum _{n = 2}^{\infty } n^2 (n - 1)^2 (n - 2) ! \langle \varphi _n (e_{\ell _1}, e_k, \cdot ), \varphi _n (e_{\ell _2}, e_k, \cdot ) \rangle _{H^{\otimes (n - 2)}_s} \langle e_{\ell _1}, e_{\ell _2} \rangle _{\dot{V}}\\&= \sum _{\ell _1, \ell _2} \sum _{n = 2}^{\infty } n^2 (n - 1) (n - 1) ! \langle \varphi _n (e_{\ell _1}, \cdot ), \varphi _n (e_{\ell _2}, \cdot ) \rangle _{H^{\otimes (n - 1)}_s} \langle e_{\ell _1}, e_{\ell _2} \rangle _{\dot{V}}\\&= \sum _{n = 1}^{\infty } (n - 1) \mathbb {E} \left[ D_{e_{\ell _1}} W_n (\varphi _n) D_{e_{\ell _2}} W_n (\varphi _n) \right] \langle e_{\ell _1}, e_{\ell _2} \rangle _{\dot{V}}\\&= \sum _{n = 1}^{\infty } \mathbb {E} \left[ D_{e_{\ell _1}} (\mathcal {N}- 1)^{1 / 2} W_n (\varphi _n) D_{e_{\ell _2}} (\mathcal {N}- 1)^{1 / 2} W_n (\varphi _n) \right] \langle e_{\ell _1}, e_{\ell _2} \rangle _{\dot{V}}\\&= \Vert \boldsymbol{1}_{\mathcal {N} \geqslant 1} (\mathcal {N}- 1)^{1 / 2} F \Vert ^2_{\dot{\mathcal {H}}^1_0} . \end{aligned}$$

$$\square $$

Our estimates for $\mathcal {G}^{\rho }_+$ and $\mathcal {G}^{\rho }_-$ are based on duality arguments, which means that we need strong assumptions on $(\rho _N)$ to obtain the strong convergence of $(\mathcal {G}^{\rho _N} F)_N$ in $\mathcal {H}^{- 1}_0$ in the next lemma. The weak convergence in $\mathcal {H}^{- 1}_0$ would require much weaker assumptions, essentially only $\rho _N h \rightarrow h$ in *H* for all $h\in H$, and $\rho _N v \rightarrow v$ in $\dot{V}$ for all $v \in \dot{V}$.

#### Lemma 2.16

(Approximation of ${\mathcal {G}} F$) Let $(\rho _N)_{N \in \mathbb {N}} \subset L (H, V)$ be such that $\sup _N \Vert \rho _N \Vert _{L (H, H)} + \Vert \rho _N \Vert _{L (\dot{V}, \dot{V})} < \infty$ and for all $f \in S$

$$\begin{aligned} \Vert (\rho _N-\text {Id}) f \Vert _V + \Vert \langle B\circ \rho _N^{\otimes 2}, f \rangle _{V^{*}, V} - \langle B, f \rangle _{V^{*}, V} \Vert _{L (V \otimes _s H, \mathbb {R})}&\leqslant C_1 (f) \varepsilon _N,\\ {\Vert \langle B (\varphi ), (\rho _N-\text {Id})\cdot \rangle _{V^{*}, V} \Vert _{L (\dot{V}, \mathbb {R})}+}\Vert \langle B (\rho _N^{\otimes 2}\varphi ), \cdot \rangle _{V^{*}, V} - \langle B (\varphi ), \cdot \rangle _{V^{*}, V} \Vert _{L (\dot{V}, \mathbb {R})}&\leqslant C_2 (\varphi ) \varepsilon _N, \end{aligned}$$

where $C_1 : S \rightarrow [0, \infty )$ and $C_2 : S \otimes _s S \rightarrow [0, \infty )$ are such that $\mathbb {E} [C_1 (D F)^2 + C_2 (D^2 F)^2] < \infty$ for all $F \in \mathcal {C}$ and where $\varepsilon _N \rightarrow 0$. Then for any cylinder function $F \in \mathcal {C}$ the limit

$$\begin{aligned} \mathcal {G}F = \lim _{N \rightarrow \infty } \mathcal {G}^{\rho _N} F \end{aligned}$$

exists in $\mathcal {H}^{- 1}_0$. The limit does not depend on the specific approximation, it satisfies the same bounds as in Lemma 2.14 with the $\rho$-dependent factors on the right hand side equal to 1, and it is also antisymmetric on cylinder functions. Moreover, for $f \in S$ we also have the convergence

43 $$\begin{aligned} \Vert \mathcal {G} (\eta (f)) - \langle {:} \tilde{B}^{\rho _N} (\eta , \eta ) {:}, f \rangle _{V^{*}, V}\Vert _{{\mathcal {H}}^{-1}_0} \lesssim \varepsilon _N C_1(f), \end{aligned}$$

where we recall that formally $\langle {:} \tilde{B}^{\rho _N} (\eta , \eta ) {:}, f \rangle _{V^{*}, V} = \langle {:} B (\rho _N \eta , \rho _N \eta ) {:}, f \rangle _{V^{*}, V}$, i.e. the approximation $\rho _N$ is not acting on *f*.

#### Proof

By similar computations as in Lemma 2.14 we obtain for $G \in \mathcal {C}$

44 $$\begin{aligned} \mathbb {E} [(\mathcal {G}_+^{\rho _N} F -\mathcal {G}^{\rho _M}_+ F) G]&= \mathbb {E} [\langle B (\rho _N^{\otimes 2}D^2 (1 +\mathcal {N})^{- 1} G) - B (\rho _M^{\otimes 2}D^2 (1 +\mathcal {N})^{- 1} G), \nonumber \\&\quad \rho _N D (2 +\mathcal {N}) F \rangle _{\dot{V}^{*}, \dot{V}}] \nonumber \\&\quad + \mathbb {E} [\langle B (\rho _M^{\otimes 2}D^2 (1 +\mathcal {N})^{- 1} G), (\rho _N - \rho _M) D (2 +\mathcal {N}) F \rangle _{\dot{V}^{*}, \dot{V}}] \nonumber \\&\lesssim \mathbb {E} [(\varepsilon _N + \varepsilon _M) \Vert D^2 (1 +\mathcal {N})^{- 1} G \Vert _{V \otimes _s H} C_1 (D (2 +\mathcal {N}) F)] \nonumber \\&\leqslant (\varepsilon _N + \varepsilon _M) \Vert G \Vert _{\mathcal {H}^1_0} \mathbb {E} [C_1 (D (2 +\mathcal {N}) F)^2]^{1 / 2} \end{aligned}$$

and analogously

$$\begin{aligned} \mathbb {E} [(\mathcal {G}_-^{\rho _N} F -\mathcal {G}^{\rho _M}_- F) G]&\lesssim \mathbb {E} [(\varepsilon _N + \varepsilon _M) C_2 (D^2 F) \Vert D G \Vert _{\dot{V}}]\\&\leqslant (\varepsilon _N + \varepsilon _M) \mathbb {E} [C_2 (D^2 F)^2]^{1 / 2} \Vert G \Vert _{\dot{\mathcal {H}}^{1}_0}, \end{aligned}$$

which shows that $(\mathcal {G}^{\rho _N} F)_N$ is Cauchy in $\mathcal {H}^{- 1}_0$ and the limit does not depend on the specific approximation, it satisfies the bounds of Lemma 2.14 and it is antisymmetric on cylinder functions.

The convergence of $\langle : \tilde{B}^{\rho _N} (\eta , \eta ):, f \rangle _{V^{*}, V} = \mathcal {G}^{\rho _N} (\eta (f)) + \langle {:} \tilde{B}^{\rho _N} (\eta , \eta ) {:}, f - \rho _N f \rangle _{V^{*}, V}$ is similar. $\square$

#### Definition 2.17

We write

45 $$\begin{aligned} \mathcal {L} := \mathcal {L}_0 + \mathcal {G} \end{aligned}$$

for the formal generator of the solution to (11). It boundedly maps $\mathcal {H}^1_\alpha$ to $\mathcal {H}^{-1}_{\alpha -1}$.

### Energy solutions

#### Definition 2.18

(Energy solution) We make Assumptions (A) and (B). Let $(u_t (f))_{t \geqslant 0, f \in S}$ be an adapted stochastic process on some filtered probability space $(\Omega , \mathcal {F}, (\mathcal {F}_t)_{t\ge 0}, \mathbb {P})$ satisfying the usual conditions. We call *u* an energy solution to the equation

$$\begin{aligned} \partial _t u = - A u + {:} B (u, u) {:} + \sqrt{2 A} \xi , \end{aligned}$$

if the following conditions are satisfied:

i. Path regularity: For all $f \in S$ the process $t \mapsto u_t (f)$ is continuous.
ii. Uniform $L^2$ bound for the density: $\sup _{t \leqslant T} | \mathbb {E} [F (u_t)] | \lesssim \Vert F \Vert _{L^2 (\mu )}$ for all $T > 0$ and $F \in {\mathcal {C}}$.
iii. Energy estimate: For all $F \in \mathcal {C}$ and for all $T > 0$
  46 $$\begin{aligned} \mathbb {E} \left[ \sup _{t \leqslant T} \left| \int _0^t F (u_s) \textrm{d}s\right| \right] \lesssim (T^{1 / 2} + T) \Vert F \Vert _{\mathcal {H}^{- 1}_0}. \end{aligned}$$
  In particular, there exists a unique continuous extension to $\mathcal {H}^{- 1}_0$ of the map
  $$ I : \mathcal {C} \rightarrow \bigcap _{T>0} L^1 (\Omega , C ([0,T], \mathbb {R})), \qquad I (F)_t = \int _0^t F (u_s) \textrm{d}s. $$
iv. Weak solution: For $f \in S$ the process
  47 $$\begin{aligned} M^f_t :=u_t (f) - u_0 (f) + \int _0^t u_s(A f) \textrm{d}s - I (\langle {:} B (\cdot , \cdot ) {:}, f \rangle _{V^{*}, V})_t, \qquad t \geqslant 0, \end{aligned}$$
  is a continuous martingale with quadratic variation $[M^f]_t = t 2 \langle A f, f \rangle _H$, and there exist $p<2$ and a sequence $(\rho _N)_{N \in {\mathbb {N}}}\subset L(H,V)$ such that for all $T>0$ the following convergence holds in probability:
  $$ \lim _{N \rightarrow \infty } \left\| I (\langle {:} B (\cdot , \cdot ) {:}, f \rangle _{V^{*}, V})_{\cdot } - \int _0^\cdot \langle {:} {\tilde{B}}^{\rho _N} (u_s, u_s) {:}, f \rangle _{V^{*}, V} \textrm{d}s \right\| _{[0,T],p-\textrm{var}} = 0, $$
  where
  $$ \Vert I \Vert _{[0,T],p-\textrm{var}} \!=\! \sup \left\{ \left( \sum _{k=0}^{n-1} |I_{t_{k+1}} - I_{t_k}|^p\right) ^{1/p}: n \in {\mathbb {N}}, 0\!=\!t_0< \dots < t_n \!=\!T \right\} . $$

In the formulation of the weak solution property iv. we used implicitly that for any $F\in L^2(\mu )$ the integral $\int _0^t F(u_s)\textrm{d}s$ makes sense by the path regularity and by the uniform $L^2$ bound for the density of $u_t$: For $F \in {\mathcal {C}}$ this follows from path regularity, and for $F \in L^2(\mu )$ we can find $(F^N) \subset \mathcal {C}$ that converges to *F* in $L^2(\mu )$, so that by the uniform $L^2$ bound for the density $(\int _0^\cdot F^N(u_s) \textrm{d}s)_N$ is Cauchy in $L^1(\Omega , C([0,T],{\mathbb {R}}))$ and we define $\int _0^\cdot F(u_s) \textrm{d}s$ as the limit (which does not depend on the approximating sequence). We also used that $\langle {:} B (\cdot , \cdot ) {:}, f \rangle _{V^{*}, V} = {\mathcal {G}} \eta (f) \in {\mathcal {H}}^{-1}_0$, which holds because $\eta (f) \in {\mathcal {H}}^1_1$.

#### Remark 2.19

The uniform $L^2$ bound for the density and the energy estimate are formulated in a way that corresponds to energy solutions that at time 0 have a distribution with density with respect to $\mu$ that is in $L^2(\mu )$. It would be possible to relax this to an $L^1$ density by replacing the control of moments by a control of probabilities, see [23, Def. 13].

In applications to scaling limits of particle systems the following characterization of energy solutions is sometimes useful.

#### Theorem 2.20

(Sufficient conditions for an energy solution) We make Assumptions (A) and (B) and additionally assume that *S* is a core for the operator *A*. Any adapted stochastic process $(u_t (f))_{t \geqslant 0, f \in S}$ that satisfies all of the following conditions is an energy solution in the sense of Definition 2.18:

i. Path regularity: For all $f \in S$ the process $t \mapsto u_t (f)$ is continuous.
ii. Stationarity: $u_t \sim \mu$ for all $t \geqslant 0$.
iii. Weak solution: There exist $p<2$ and a sequence $(\rho _N)_{N \in {\mathbb {N}}}\subset L(H,V)$ such that for all $f \in S$ there is a process ${\mathcal {B}}^f$ with
  $$\begin{aligned} \lim _{N \rightarrow \infty }\mathbb {P}\left( \left\| {\mathcal {B}}^f - \int _0^\cdot \langle {:} {\tilde{B}}^{\rho _N} (u_s, u_s) {:}, f \rangle _{V^{*}, V} \textrm{d}s \right\| _{[0,T],p-\textrm{var}}>\varepsilon \right)&\!=\! 0, \qquad \varepsilon , T\!>\!0,\\ \lim _{N\rightarrow \infty } \left\| \langle {:} B (\eta , \eta ) {:}, f \rangle _{V^{*}, V} - \langle {:} {\tilde{B}}^{\rho _N} (\eta , \eta ) {:}, f \rangle _{V^{*}, V}\right\| _{\mathcal {H}^{-1}_0}&= 0. \end{aligned}$$
  Moreover, the process
  $$\begin{aligned} M^f_t :=u_t (f) - u_0 (f) + \int _0^t u_s(Af)\textrm{d}s - {\mathcal {B}}^f_t, \qquad t \geqslant 0, \end{aligned}$$
  is a continuous martingale with quadratic variation $[M^f]_t = 2t \langle A f, f \rangle _H$.
iv. Behavior under time-reversal: for $T > 0$ the processes $\hat{u}_t (f) = u_{T - t} (f)$, and $\hat{{\mathcal {B}}}^f_t = ({\mathcal {B}}^f_T - {\mathcal {B}}^f_{T-t})$, $t \in [0, T]$, are such that
  $$ \hat{M}^f_t :=\hat{u}_t (f) - \hat{u}_0 (f) + \int _0^t \hat{u}_s(Af)\textrm{d}s + \hat{{\mathcal {B}}}^f_t, \qquad t \in [0,T], $$
  is a continuous martingale in the filtration generated by $\hat{u}$, with quadratic variation $[\hat{M}^f]_t = 2 t \langle A f, f \rangle _H$.

If the sequence $(\rho _N)_{N\in {\mathbb {N}}}$ is as in Assumption (B), then in point iii. it suffices to verify uniform convergence on compacts of $\int _0^\cdot \langle {:} {\tilde{B}}^{\rho _N} (u_s, u_s) {:}, f \rangle _{V^{*}, V} \textrm{d}s$ to ${\mathcal {B}}^f$ and that ${\mathcal {B}}^f$ is of vanishing quadratic variation. It is not necessary to verify the convergence of $\langle {:} {\tilde{B}}^{\rho _N} (\eta , \eta ) {:}, f \rangle _{V^{*}, V}$ to $\langle {:} B (\eta , \eta ) {:}, f \rangle _{V^{*}, V}$.

#### Proof

There are two statements which are not obvious. First, that i.-iv. imply an improved version of the energy estimate, which is shown in Lemma 2.21 below, and which establishes that *u* is an energy solution. The lemma does not actually require the full strength of iii., and in particular the only requirement on ${\mathcal {B}}^f$ is that it is of vanishing quadratic variation.

Lemma 2.21 then allows us to show the second claim: Assume that the sequence $(\rho _N)_{N\in {\mathbb {N}}}$ is as in Assumption (B). Then the convergence of $\langle {:} {\tilde{B}}^{\rho _N} (\eta , \eta ) {:}, f \rangle _{V^{*}, V}$ to $\langle {:} B (\eta , \eta ) {:}, f \rangle _{V^{*}, V}$ in $\mathcal {H}^{-1}_0$ follows from Lemma 2.16. If in point iii. the process ${\mathcal {B}}^f$ is of vanishing quadratic variation and we have uniform convergence on compacts of $\int _0^\cdot \langle {:} {\tilde{B}}^{\rho _N} (u_s, u_s) {:}, f \rangle _{V^{*}, V} \textrm{d}s$ to ${\mathcal {B}}^f$, we obtain the improved energy estimate (49) by Lemma 2.21. It remains to show that the convergence

$$ \int _0^t\cdot \langle {:} {\tilde{B}}^{\rho _N} (u_s, u_s) {:}, f \rangle _{V^{*}, V} \textrm{d}s \rightarrow {\mathcal {B}}^f $$

holds in *p*-variation for some $p<2$. By the Besov-variation embedding shown in Corollary A.3 of the appendix of [14] it suffices to show that, with $I_{s,t} = I_t - I_s$ and for some $p<2$,

48 $$\begin{aligned} \lim _{N\rightarrow \infty } \int _{[0,T]^2}\frac{{\mathbb {E}}[|I(\langle {:} {\tilde{B}}^{\rho _N} (\cdot , \cdot ) {:}, f \rangle _{V^{*}, V} - \langle {:} B (\cdot , \cdot ) {:}, f \rangle _{V^{*}, V})_{s,t}|^2]}{|t-s|^{1+2/p}}\textrm{d}s \textrm{d}t =0. \end{aligned}$$

Shifting (49) to the interval [*s*, *t*] by stationarity and ignoring the higher order contribution $(t-s)^2$ because we are interested in short length scales, we obtain from the improved energy estimate and (43) for all $M \in {\mathbb {N}}$

$$ {\mathbb {E}}[|I(\langle {:} {\tilde{B}}^{\rho _M} (\cdot , \cdot ) {:}, f \rangle _{V^{*}, V} - \langle {:} B (\cdot , \cdot ) {:}, f \rangle _{V^{*}, V})_{s,t}|^2] \lesssim \varepsilon _M |t-s|. $$

Let $\kappa = \tfrac{1}{1+\alpha }$, where $\Vert \rho _N\Vert _{L(H,V)} \lesssim \varepsilon _N^{-\alpha }$. If $\varepsilon _N \leqslant |t-s|^\kappa$, we take $M=N$. If $\varepsilon _N > |t-s|^\kappa$ we take $M>N$ such that $\varepsilon _M \simeq |t-s|^\kappa$ and obtain from (49) together with (19), writing $I, I^N, I^M$ for brevity,

$$\begin{aligned} {\mathbb {E}}[|I_{s,t} - I^N_{s,t}|^2]&\lesssim {\mathbb {E}}[|I_{s,t} - I^M_{s,t}|^2] + {\mathbb {E}}[|I^N_{s,t}|^2] + {\mathbb {E}}[|I^M_{s,t}|^2] \lesssim \varepsilon _M |t-s|\\&\quad + \varepsilon _M^{-\alpha }|t-s|^2 \simeq |t-s|^{1+\kappa }. \end{aligned}$$

Interpolating this with the bound ${\mathbb {E}}[|I_{s,t} - I^N_{s,t}|^2] \lesssim \varepsilon _N |t-s|$, we obtain that (48) holds for all $p>2/(1+\kappa )$. $\square$

The proof used the following lemma, which might be useful in its own right.

#### Lemma 2.21

We make Assumption (A) and additionally assume that *S* is a core for *A*. Let $(u_t (f))_{t \geqslant 0, f \in S}$ be an adapted process which satisfies conditions i., ii., iv. from Theorem 2.20 and also

iii.’ For each $f\in S$ there exists a process ${\mathcal {B}}^f$ of vanishing quadratic variation such that
  $$\begin{aligned} M^f_t :=u_t (f) - u_0 (f) + \int _0^t u_s(Af)\textrm{d}s - {\mathcal {B}}^f_t, \qquad t \geqslant 0, \end{aligned}$$
  is a continuous martingale with quadratic variation $[M^f]_t = 2t \langle A f, f \rangle _H$.

Then *u* satisfies the improved energy estimate

49 $$\begin{aligned} \mathbb {E} \left[ \sup _{t \leqslant T} \left| \int _0^t F (u_s) \textrm{d}s \right| ^2 \right] \lesssim (T + T^2) \Vert F \Vert _{\mathcal {H}^{- 1}_0}^2,\qquad F\in {\mathcal {C}}. \end{aligned}$$

#### Proof

For $F \in \mathcal {C}$ we obtain from the Itô trick (we can apply Itô’s formula for Dirichlet processes by the assumption that the drift has vanishing quadratic variation, together with the usual time reversal argument as in [20]) that

50 $$\begin{aligned} \mathbb {E} \left[ \sup _{t \leqslant T} \left| \int _0^t \mathcal {L}_0 F (u_s) \textrm{d}s \right| ^2 \right] \lesssim T \Vert F \Vert _{\mathcal {H}^1_0}^2. \end{aligned}$$

By Lemma A.4 in the appendix, $\mathcal {C}$ is a core for $\mathcal {L}_0$. Therefore, the bound (50) extends to all $F \in \mathcal {D} (\mathcal {L}_0)$: Let $(F^N)_N \subset \mathcal {C}$ be such that $F^N \rightarrow F$ and $\mathcal {L}_0 F^N \rightarrow \mathcal {L}_0 F$. Then also

$$ \Vert F - F^N \Vert _{\mathcal {H}^1_0}^2 \simeq \mathbb {E} [(F - F^N) (1 -\mathcal {L}_0) (F - F^N)] \leqslant \Vert F - F^N \Vert \cdot \Vert (1 -\mathcal {L}_0) (F - F^N) \Vert \rightarrow 0, $$

and thus by stationarity

$$\begin{aligned} \mathbb {E} \left[ \sup _{t \leqslant T} \left| \int _0^t \mathcal {L}_0 F (u_s) \textrm{d}s \right| ^2 \right]&= \lim _{N \rightarrow \infty } \mathbb {E} \left[ \sup _{t \leqslant T} \left| \int _0^t \mathcal {L}_0 F^N (u_s) \textrm{d}s \right| ^2 \right] \lesssim \lim _{N \rightarrow \infty } T \Vert F^N \Vert _{\mathcal {H}^1_0}^2 \\&= T \Vert F \Vert _{\mathcal {H}^1_0}^2 . \end{aligned}$$

Let now $F \in {\mathcal {C}}$ and let $(1 -\mathcal {L}_0) G = F$, so $G \in \mathcal {D} (\mathcal {L}_0)$. Then

$$\begin{aligned} \mathbb {E} \left[ \sup _{t \leqslant T} \left| \int _0^t F (u_s) \textrm{d}s \right| ^2 \right]&\lesssim \mathbb {E} \left[ \sup _{t \leqslant T} \left| \int _0^t G (u_s) \textrm{d}s \right| ^2 \right] +\mathbb {E} \left[ \sup _{t \leqslant T} \left| \int _0^t \mathcal {L}_0 G (u_s) \textrm{d}s \right| ^2 \right] \\&\lesssim T^2 \Vert G \Vert ^2 + T \Vert G \Vert _{\mathcal {H}^1_0}^2, \end{aligned}$$

and clearly $\Vert G \Vert \leqslant \Vert G \Vert _{\mathcal {H}^1_0}$, while $\Vert G \Vert _{\mathcal {H}^{1}_0} \leqslant \Vert F \Vert _{\mathcal {H}^{- 1}_0}$ because

$$ \Vert G \Vert _{\mathcal {H}^1_0}^2 \simeq \mathbb {E} [G (1 -\mathcal {L}_0) G] =\mathbb {E} [G F] \leqslant \Vert G \Vert _{\mathcal {H}^1_0} \Vert F \Vert _{\mathcal {H}^{- 1}_0}. $$

This proves the claimed energy estimate (49). $\square$

## Energy solutions and semigroup

### Construction of the semigroup and resolvent

#### Proposition 3.1

Let $\mathcal {G}^N = \boldsymbol{1}_{\mathcal {N} \leqslant N} \mathcal {G} \boldsymbol{1}_{\mathcal {N} \leqslant N}$. The following statements hold:

1. For every $N{\in }\mathbb {N}$ and $F^{\sharp } \in \mathcal {H}^{- 1}_0$ there is a unique solution $F^N =:(1 - \mathcal {L}_0 - \mathcal {G}^N)^{- 1} F^{\sharp } \in \mathcal {H}^1_0$ to the resolvent equation
  51 $$\begin{aligned} (1 - \mathcal {L}_0 - \mathcal {G}^N) F^N = F^{\sharp } . \end{aligned}$$
2. Furthermore, the following bound holds uniformly in *N*
  52 $$\begin{aligned} {\Vert (1 - \mathcal {L}_0 - \mathcal {G}^N)^{- 1} F^{\sharp } \Vert _{\mathcal {H}_{ 0}^1}} \lesssim {\Vert F^{\sharp } \Vert _{\mathcal {H}_{ 0}^{- 1}}} . \end{aligned}$$
3. There exists $\mathcal {R}_1 \in L (\mathcal {H}^{- 1}_0, \mathcal {H}^1_0)$ such that for every $F^{\sharp } \in \mathcal {H}^{- 1}_0$
  53 $$\begin{aligned} (1 - \mathcal {L}_0 - \mathcal {G}^N)^{- 1} F^{\sharp } \rightarrow \mathcal {R}_1 F^{\sharp }, \end{aligned}$$
  weakly in $\mathcal {H}^1_0$ and $F = \mathcal {R}_1 F^{\sharp } \in \mathcal {H}^1_0$ is a solution to the resolvent equation
  $$\begin{aligned} (1 - \mathcal {L}_0 - \mathcal {G}) F = F^{\sharp } . \end{aligned}$$
4. With $\mathcal {D} = \mathcal {R}_1 L^2 (\mu ) \subset \mathcal {D}_{\max } :=\{ F \in \mathcal {H}^1_0 : \mathcal {L} F \in L^2 (\mu ) \} \subset \mathcal {H}^1_0$ it holds that $(\mathcal {D}, \mathcal {L})$ generates a strongly continous contraction semigroup $(P_t)_{t \geqslant 0}$ on $L^2 (\mu )$.

#### Proof

1. We want to apply the Lax-Milgram theorem, see [13, sec. 6.21]. For this purpose, we consider the bilinear operator $K^N : \mathcal {H}^1_0 \times \mathcal {H}^1_0 \rightarrow \mathbb {R}$ given by
  54 $$\begin{aligned} K^N (\cdot , \cdot ) :=\langle (1 -\mathcal {L}_0 -\mathcal {G}^N) \cdot , \cdot \rangle _{ \mathcal {H}^{- 1}_0,\mathcal {H}^1_0} . \end{aligned}$$
  By Lemmas 2.12 and 2.14 (bounds on $\mathcal {L}_0$ and $\mathcal {G}$) the operator $(1 -\mathcal {L}_0 -\mathcal {G}^N)$ continuously maps $\mathcal {H}^1_0$ to $\mathcal {H}^{- 1}_0$ and
  55 $$\begin{aligned} K^N (F, G)&= \langle (1 -\mathcal {L}_0) F, \mathcal {} G \rangle _{\mathcal {H}^1_0, \mathcal {H}^{- 1}_0 } - \langle \mathcal {G}^N F, \mathcal {} G \rangle _{\mathcal {H}^1_0, \mathcal {H}^{- 1}_0} \nonumber \\&\leqslant \Vert F \Vert _{\mathcal {H}^1_0} \Vert G \Vert _{\mathcal {H}^1_0} +\Vert \mathcal {G}^N F\Vert _{\mathcal {H}^1_0} \Vert \mathcal {} G\Vert _{\mathcal {H}^{- 1}_0 } \nonumber \\&\lesssim _N (\Vert F \Vert _{\mathcal {H}^1_0} + \Vert \boldsymbol{1}_{\mathcal {N} \leqslant n} F \Vert _{\mathcal {H}^1_0}) \Vert G \Vert _{\mathcal {H}^1_0} \nonumber \\&\lesssim \Vert F \Vert _{\mathcal {H}^1_0} {\Vert G \Vert _{\mathcal {H}^1_0}} . \end{aligned}$$
  Moreover, by Lemma 2.12 (bounds on $\mathcal {L}_0$) and the fact that $\mathcal {G}^N$ is antisymmetric, we have for every $F \in \mathcal {H}^1_0 \mathcal {}$
  56 $$\begin{aligned} K^N (F, F)&= \langle (1 -\mathcal {L}_0 -\mathcal {G}^N) F, \mathcal {} F \rangle _{\mathcal {H}^1_0, \mathcal {H}^{- 1}_0} \nonumber \\&= \langle (1 -\mathcal {L}_0) \mathcal {} F, F \rangle _{\mathcal {H}^1_0, \mathcal {H}^{- 1}_0} \nonumber \\&\simeq \Vert F \Vert _{\mathcal {H}^1_0}^2 . \end{aligned}$$
  Therefore, $K^N$ satisfies the conditions of the Lax-Milgram theorem, and given $F^{\sharp } \in \mathcal {H}^{- 1}_0$, there exists a unique $F^N \in \mathcal {H}^1_0$ such that
  57 $$\begin{aligned} K^N (F^N, G) = \langle F^{\sharp }, G \rangle _{\mathcal {H}^{- 1}_0, \mathcal {H}^1_0}, \qquad G \in \mathcal {H}^1_0 . \end{aligned}$$
  This concludes the proof of 1.
2. By eq. (56) we have
  58 $$\begin{aligned} \Vert F^{\sharp } \Vert _{\mathcal {H}^{- 1}_0} \Vert F^N \Vert _{\mathcal {H}^1_0} \geqslant \langle F^{\sharp }, F^N \rangle _{\mathcal {H}^{- 1}_0, \mathcal {H}^1_0} = K^N (F^N, F^N ) \simeq \Vert F^N \Vert _{\mathcal {H}^1_0}^2, \end{aligned}$$
  uniformly in *N*, so that
  59 $$\begin{aligned} \Vert F^N \Vert _{\mathcal {H}^1_0} \lesssim \Vert F^{\sharp } \Vert _{\mathcal {H}^{- 1}_0} . \end{aligned}$$
3. With the weak convergence, this follows by testing against $G \in \mathcal {C}$ and applying the dominated convergence theorem and using that $G \in \mathcal {H}^1_1$ to obtain $\Vert (\mathcal {G} - \mathcal {G}^N) G \Vert _{\mathcal {H}^{- 1}_0} \rightarrow 0$. See [33, Prop. 1.2] or [22, Prop. 4.9] for details.
4. This is due to [24] and it follows from the Lumer-Pillips theorem. See [33, Prop. 1.2] or [22, Prop. 4.9] for details.$\square$

#### Lemma 3.2

It holds that

$$ \langle (1 -\mathcal {L}) F, F \rangle _{L^2 (\mu )} \simeq \Vert F \Vert _{\mathcal {H}^1_0}^2, \qquad F \in \mathcal {D}_{\max } = \{ F \in \mathcal {H}^1_0 : \mathcal {L} F \in L^2 (\mu ) \} \subset \mathcal {H}^1_0 . $$

Consequently, $(1 -\mathcal {L})$ is injective on $\mathcal {D}_{\max }$,

$$\begin{aligned} (1 - \mathcal {L}) \mathcal {C} \subset \mathcal {H}^{- 1}_0, \end{aligned}$$

is dense in $\mathcal {H}^{- 1}_0$ and the domain $\mathcal {D}$ from the previous Proposition 3.1 is equal to the maximal domain $\mathcal {D}_{\max }$.

#### Proof

The proof is based on the commutator estimate (30) and can be found in e.g. [33, Thm. 1.4] or [22, Thm. 4.11]. $\square$

### Construction of energy solutions

In this section we assume that there exists a complete orthonormal system $(e_n)_{n \in \mathbb {N}} \subset V$ with respect to *H* of eigenvectors for *A* with an increasing sequence of eigenvalues $(\lambda _n)_{n \in \mathbb {N}}$, which morally corresponds to the case that *V* is compactly embedded in *H*. This assumption could be bypassed by a more complex approximation argument, involving a natural discretization of the spectrum of *A* that leads to an *H*-bounded perturbation of this operator, but for simplicity we restrict our attention to the special case where *A* can be diagonalized. Note that $\lambda _n \geqslant 0$ for all $n \in \mathbb {N}$ because $\langle A f, f \rangle _H \geqslant c \Vert f \Vert _{\dot{V}}^2$. We also assume that *S* is a nuclear Fréchet space.

Let $N \in \mathbb {N}$. Consider the projected SDE corresponding to equation (11), for $k = 1, \ldots , N$,

60 $$\begin{aligned} \textrm{d}u^N_t (e_k) = \left( - \lambda _k u^N_t (e_k) + \langle {:}B^N(u^N_t,u^N_t){:}, e_k\rangle _{V^*, V} \right) \textrm{d}t + \sqrt{2 \lambda _k} \textrm{d}W^k_t, \end{aligned}$$

where $(W^k)_k$ are i.i.d. Brownian motions and

$$ {:}B^N (u, u){:} :=\sum _{k, \ell , m \leqslant N} (u (e_{\ell }) u (e_m) - \delta _{\ell , m}) \langle B (e_{\ell }, e_m), e_k \rangle _{V^{*}, V} e_k. $$

We claim that an invariant probability measure is

$$ (u^N_k)^N_{k = 1} : = (u^N (e_k))_{k = 1}^N \sim \mu _N =\mathcal {N} (0,\mathbb {I}_{N \times N}) . $$

Indeed, the generator of the SDE is

$$ \mathcal {L}^N = \sum _{k = 1}^N \left( \lambda _k (- u^N_k \partial _k + \partial _{k k}) + \sum _{\ell , m \leqslant N} (u^N_{\ell } u^N_m - \delta _{\ell , m}) \langle B (e_{\ell }, e_m), e_k \rangle _{V^{*}, V} \partial _k \right) . $$

It can be split up into a symmetric part

$$ \mathcal {L}_0^N = \sum _{k = 1}^N \lambda _k (- u^N_k \partial _k + \partial _{k k}), $$

which is the generator of an Ornstein-Uhlenbeck process in *N* dimensions and hence preserves $\mu _N$, whereas by analogous reasoning as in Lemma 2.7 the contribution of the drift

$$ \mathcal {G}^N = \sum _{k, \ell , m \leqslant N} (u^N_{\ell } u^N_m - \delta _{\ell , m}) \langle B (e_{\ell }, e_m), e_k \rangle _{V^{*}, V} \partial _k, $$

is antisymmetric and hence the full generator $\mathcal {L}^N$ has $\mu _N$ as an invariant measure. We start $u^N$ in its invariant measure (and discuss relaxed assumptions later), and we test against $f \in H$ via

$$\begin{aligned} u^N_t (f) = \sum _{k = 1}^N u^N_t (e_k) \langle e_k, f \rangle _H . \end{aligned}$$

Let us first derive an energy estimate for $u^N$, uniformly in *N*. We define the projection

61 $$\begin{aligned} \Pi _N h = \sum _{k=1}^N \langle h,e_k\rangle _H e_k, \end{aligned}$$

and for a cylinder function $F(\eta )=\Phi (\eta (f))$ we define a new cylinder function $\Pi _N F(\eta ) = \Phi (\eta (\Pi _N f))$.

#### Lemma 3.3

(Itô trick and energy estimate) For any $p \geqslant 2$ the following “Itô trick” bound holds uniformly in *N*

62 $$\begin{aligned} \mathbb {E} \left[ \sup _{t \leqslant T} \left| \int _0^t \mathcal {L}_0^N F (u_s^N) \textrm{d}s \right| ^p \right] \lesssim T^{p / 2} \left\| \sum _{k = 1}^N \lambda _k | \partial _k F |^2 \right\| _{L^{p / 2} (\mu _N)}^{p / 2}, \qquad F \in \mathcal {C}, \end{aligned}$$

and if $p = 2$ or if $F(\eta ) = \Phi (\eta (f))$ with a polynomial $\Phi$ of degree *M*, then we also have the *energy estimate*

63 $$\begin{aligned} \mathbb {E} \left[ \sup _{t \leqslant T} \left| \int _0^t F (u_s^N) \textrm{d}s \right| ^p \right] \lesssim (T^{p / 2} + T^p) \Vert \Pi _N F \Vert _{\mathcal {H}^{- 1}_0}^p, \end{aligned}$$

with an implicit constant that depends on *M* if $p > 2$.

#### Proof

We assume without loss of generality that $F=\Pi _N F$; otherwise we use that $F=\Pi _N F$ $\mu ^N$-a.s. and derive the estimate for $\Pi _N F$ in place of *F*. By time reversal of Markov processes, $u^N$ is an energy solution for the projected equation (60) with non-linearity $B^N$ in the sense of Lemma 2.20. Therefore, the same arguments as in [20, Proposition 3.2] show that for any cylinder function *F*

$$ 2 \int _0^t \mathcal {L}_0^N F (u_s^N) \textrm{d}s = M_t^F + \hat{M}^F_{T - t} - \hat{M}^F_T, $$

where $\textrm{d}M^F_t = \sum _{k = 1}^N \partial _k F (u^N_t) \sqrt{2 \lambda _k} \textrm{d}W^k_t$ and thus $\textrm{d}[M^F]_t = 2 \sum _{k = 1}^N \lambda _k | \partial _k F (u^N_t) |^2,$ and similarly for the backward martingale $\hat{M}^F$. The Burkholder-Davis-Gundy inequality now yields

$$ \mathbb {E} \left[ \sup _{t \leqslant T} \left| \int _0^t \mathcal {L}_0^N F (u_s^N) \textrm{d}s \right| ^p \right] \lesssim T^{p / 2} \left\| \sum _{k = 1}^N \lambda _k | \partial _k F |^2 \right\| _{L^{p / 2} (\mu _N)}^{p / 2}, $$

and for $p = 2$ the right hand side is

$$ T \sum _{k = 1}^N \lambda _k \Vert \partial _k F \Vert _{L^2 (\mu _N)}^2 = T \langle F, (-\mathcal {L}_0^N) F \rangle _{L^2 (\mu ^N)} . $$

If $\Phi$ is a polynomial of degree *M*, then $\sum _{k = 1}^N \lambda _k | \partial _k F |^2$ is a polynomial of degree $2 M - 2$ and thus we obtain from Gaussian hypercontractivity

$$ \left\| \sum _{k = 1}^N \lambda _k | \partial _k F |^2 \right\| _{L^{p / 2} (\mu _N)}^{p / 2} \simeq _M \langle F, (-\mathcal {L}_0^N) F \rangle _{L^2 (\mu ^N)}^{p/2}. $$

We can of course also bound $\mathbb {E} \left[ \sup _{t \leqslant T} \left| \int _0^t F (u_s^N) \textrm{d}s \right| ^p \right] \lesssim T^p \Vert F \Vert _{L^p (\mu _N)}^p$ with the triangle inequality, and this means that for $p = 2$ or in the hypercontractive case

$$ \mathbb {E} \left[ \sup _{t \leqslant T} \left| \int _0^t (1 -\mathcal {L}_0^N) F (u_s^N) \textrm{d}s \right| ^p \right] \lesssim (T^{p / 2} + T^p) \Vert (1 -\mathcal {L}_0^N)^{1 / 2} F \Vert _{L^2 (\mu ^N)}^p, $$

or, noting that we can invert $(1 -\mathcal {L}^N_0)$ in $L^2(\mu ^N)$,

$$\begin{aligned} \mathbb {E} \left[ \sup _{t \leqslant T} \left| \int _0^t F (u_s^N) \textrm{d}s \right| ^p \right]&\lesssim (T^{p / 2} + T^p) \Vert (1 -\mathcal {L}_0^N)^{1 / 2} (1 -\mathcal {L}_0^N)^{- 1} F \Vert _{L^2 (\mu ^N)}^p\\&= (T^{p / 2} + T^p) \Vert (1 -\mathcal {L}_0^N)^{- 1 / 2} F \Vert _{L^2 (\mu ^N)}^p \end{aligned}$$

By duality and density of $\Pi _N {\mathcal {C}}$ in $L^2(\mu ^N)$, we have for $\eta ^N\sim \mu ^N = \mu \circ \Pi _N^{-1}$

$$\begin{aligned} \Vert (1 -\mathcal {L}_0^N)^{- 1 / 2} F \Vert _{L^2 (\mu ^N)} = \sup \{ \mathbb {E} [F (\eta ^N) G (\eta ^N)] : G = \Pi _N G \in \mathcal {C}, \Vert (1 -\mathcal {L}_0^N)^{1 / 2} G \Vert _{L^2 (\mu ^N)} \leqslant 1 \}. \end{aligned}$$

We use that $\mathcal {L}_0^N G = \mathcal {L}_0 G = \Pi _N \mathcal {L}_0 G$ because $e_k$ are eigenfunctions of *A* to obtain

$$ \Vert (1 -\mathcal {L}_0^N)^{1 / 2} G \Vert _{L^2 (\mu ^N)} = \Vert (1 -\mathcal {L}_0)^{1 / 2} G \Vert _{L^2 (\mu )}, $$

and since $F=\Pi _N F$ and $G=\Pi _N G$, we end up with

$$\begin{aligned} \Vert (1 -\mathcal {L}_0^N)^{- 1 / 2} F \Vert _{L^2 (\mu ^N)}&= \sup \{ \mathbb {E} [F (\eta ^N) G (\eta ^N)] : G \in \mathcal {C}, G \\&= \Pi _N G, \Vert (1 -\mathcal {L}_0)^{1 / 2} G \Vert _{L^2 (\mu )} \leqslant 1 \}\\&\leqslant \sup \{ \mathbb {E} [ F (\eta ) G (\eta )] : G \in \mathcal {C}, \Vert (1 -\mathcal {L}_0)^{1 / 2} G \Vert _{L^2 (\mu )} \leqslant 1 \}\\&= \Vert (1 -\mathcal {L}_0)^{- 1 / 2} F \Vert _{L^2 (\mu )} . \end{aligned}$$

$$\square $$

#### Lemma 3.4

For all $f \in V$ the processes $(u^N (f))_{N \in \mathbb {N}}$ are uniformly tight in $C({\mathbb {R}}_+, {\mathbb {R}})$, and there exists $p<2$ such that also the real-valued random variables $(\Vert \int _0^\cdot \langle {:}B^N (u^N_r, u^N_r){:}, f \rangle _{V^{*}, V} \textrm{d}r \Vert _{[0,T],p-var})_{N}$ are uniformly tight for all $T>0$.

#### Proof

Tightness at the initial time is clear, because

$$\begin{aligned} \mathbb {E} [u^N_0 (f)^2] = \Vert \Pi _N f \Vert _H^2 \leqslant \Vert f \Vert _H^2 . \end{aligned}$$

For the time increment $u^N_{s,t}(f) = u^N_t(f) - u^N_s(f)$ we obtain

$$\begin{aligned} u^N_{s, t} (f)&= \int _s^t u^N_r (- A \Pi _N f) \textrm{d}r + \int _s^t \langle {:}B^N (u^N_r, u^N_r){:}, f \rangle _{V^{*}, V} \textrm{d}r + M^{f, N}_{s, t}. \end{aligned}$$

The quadratic variation of $M^{f,N}$ is

$$\begin{aligned}&[M^{f, N}]_{s,t} = 2 (t-s) \sum _{k = 1}^N \lambda _k \langle f, e_k \rangle _H^2 \leqslant 2 (t-s) \sum _{k = 1}^\infty \lambda _k \langle f, e_k \rangle _H^2\\&\quad = 2 (t-s) \langle A f, f \rangle _H \simeq (t-s) \Vert f \Vert _{\dot{V}}^2, \end{aligned}$$

which gives tightness for the martingale. Also, $u (A \Pi _N f) =\mathcal {L}_0^N u (f)$ and thus by the Itô trick (62)

$$\begin{aligned}&\mathbb {E} \left[ \left| \int _s^t u^N_r (- A \Pi _N f) \textrm{d}r \right| ^p \right] \lesssim | t - s |^{p / 2} \sum _{k = 1}^N \lambda _k \langle f, e_k \rangle _H^2 \\&\quad \leqslant | t - s |^{p / 2} \Vert f \Vert _{\dot{V}}^2, \end{aligned}$$

which yields tightness for the linear contribution to the drift. It remains to treat the nonlinear part of the drift. Note that $\langle {:}B^N(\eta ,\eta ){:}, f\rangle = {\mathcal {G}}^{\Pi _N} \eta (f)$ is a second order polynomial in $\eta$, and therefore the hypercontractive version of the energy estimate, (63), together with the bounds for $\mathcal {G}^{\Pi _N}$ from (39) yields

64 $$\begin{aligned}&\mathbb {E}\left[ \left| \int _s^t\langle {:}B^N(u^N_r,u^N_r){:},\Pi _Nf\rangle _H\textrm{d}r\right| ^p\right] \nonumber \\&\quad \lesssim |t-s|^{p/2}\left\| {\mathcal {G}}^{\Pi _N} \eta (f)\right\| _{\mathcal {H}^{-1}_0}^2 \nonumber \\&\quad \lesssim |t-s|^{p/2}(\Vert \Pi _N \Vert _{L (H, H)} + \Vert \Pi _N \Vert _{L (\dot{V}, \dot{V})})^3 \Vert \eta (f) \Vert _{\mathcal {H}^1_{1}} \nonumber \\&\quad \simeq |t-s|^{p/2}(\Vert \Pi _N \Vert _{L (H, H)} + \Vert \Pi _N \Vert _{L (\dot{V}, \dot{V})})^3 \Vert f \Vert _{V}, \end{aligned}$$

uniformly in *N*. Thus, we obtain tightness as soon as we show that $\Vert \Pi _N \Vert _{L (H, H)} + \Vert \Pi _N \Vert _{L (\dot{V}, \dot{V})}\lesssim 1$. The first term is clearly bounded by 1 because $\Pi _N$ is an orthogonal projection in *H*. For the second term we use that $(e_k)$ are eigenvectors for *A* to bound

$$ \Vert \Pi _N f \Vert _{\dot{V}}^2 \simeq \langle A \Pi _N f, \Pi _N f\rangle _{V^*, V} \leqslant \langle A f, f\rangle _{V^*, V} \simeq \Vert f\Vert _{\dot{V}}^2, $$

and this concludes the proof of tightness of $(u^N)$. Tightness for the *p*-variation is shown with the same argument as in Lemma 2.20. $\square$

#### Theorem 3.5

(Existence of energy solutions) We make Assumption 1 and 1 and we assume that there exists an orthonormal basis of eigenvectors for *A* and that *S* is a nuclear Fréchet space. Let $\nu \ll \mu$ be such that $\frac{\textrm{d}\nu }{\textrm{d}\mu } \in L^2(\mu )$. Then there exists an energy solution *u* with $u_0 \sim \nu$.

#### Proof

Let ${\mathcal {F}}_N = \sigma (\eta (e_1), \dots , \eta (e_N))$ and let $\gamma ^N = \mathbb {E}[\textrm{d}\nu / \textrm{d}\mu |{\mathcal {F}}_N]$ (conditional expectation in $L^2(\mu )$) and $\textrm{d}\nu ^N = \gamma ^N \textrm{d}\mu ^N$. Let $u^N$ be a solution to the SDE (60) with $u^N_0\sim \nu ^N$. We claim that the sequence $(u^N)_{N \in {\mathbb {N}}}$ is uniformly tight in $C({\mathbb {R}}_+, S')$ and that any limit point *u* is an energy solution with $u_0 \sim \nu$.

To show tightness of $(u^N)_N$ in $C({\mathbb {R}}_+, S')$ we apply Mitoma’s criterion [31], which states that it suffices to show tightness of $(u^N(f))_N$ in $C({\mathbb {R}}_+, {\mathbb {R}})$ for all $f \in S$; this is the only part of the argument where we use that *S* is a nuclear Fréchet space. Lemma 3.4 gives us the tightness of $(u^N(f))_N$ if $\nu =\mu$. Otherwise, we perform a change of measure at the initial time and we apply the Cauchy-Schwarz inequality and Jensen’s inequality: For any bounded and measurable $\Psi : C({\mathbb {R}}_+, {\mathbb {R}}) \rightarrow \mathbb {R}$

65 $$\begin{aligned} \mathbb {E}_{\nu ^N}[\Psi (u^N)]&= \int \frac{\textrm{d}\nu ^N}{\textrm{d}\mu ^N}(u) \mathbb {E}_{u}[\Psi (u^N)] \mu ^N(\textrm{d}u)\nonumber \\&\leqslant \left\| \frac{\textrm{d}\nu ^N}{\textrm{d}\mu ^N} \right\| _{L^2(\mu ^N)} \left( \int \mathbb {E}_{u}[\Psi (u^N)]^2 \mu ^N(\textrm{d}u)\right) ^{1/2} \nonumber \\&\leqslant \left\| \frac{\textrm{d}\nu }{\textrm{d}\mu } \right\| _{L^2(\mu )} \mathbb {E}_{\mu ^N}[\Psi (u^N)^2]^{1/2}. \end{aligned}$$

Tightness follows by taking $\Psi$ as the indicator function of the complement of a compact set *K* in $C({\mathbb {R}}_+, {\mathbb {R}})$ such that $\sup _N \mathbb {P}_{\mu ^N}(u^N(f) \in K^c) \leqslant \varepsilon$.

Next, we fix a limit point *u* along a subsequence that we denote for simplicity again with $(u_N)$. We need to show that *u* is an energy solution. Taking $\Psi (u) = |F(u_t)|$ in (65), we obtain the uniform incompressibility estimate

$$\begin{aligned} \mathbb {E}_{\nu ^N}[|F(u^N_t)|] \leqslant \left\| \frac{\textrm{d}\nu }{\textrm{d}\mu } \right\| _{L^2(\mu )} \left\| \Pi _N F \right\| _{L^2(\mu )}. \end{aligned}$$

Taking $\Psi (u) = \sup _{t \leqslant T}| \int _0^t F(u_s)\textrm{d}s|$ and combining (65) with the energy estimate (63) gives

$$ \mathbb {E}_{\nu ^N}\left[ \sup _{t \leqslant T}\left| \int _0^t F(u^N_s) \textrm{d}s\right| \right] \lesssim (T^{1/2}+T) \Vert \Pi _N F\Vert _{{\mathcal {H}}^{-1}_0}. $$

By Fatou’s lemma, *u* satisfies points i. (path regularity), ii. (incompressibility), iii. (energy estimate) in Definition 2.18 of energy solutions, provided that in ii. and iii. we restrict to cylinder functions satisfying $F = \Pi _N F$ for some $N \in \mathbb {N}$. Since such “finite range” cylinder functions are closed under pointwise multiplication, the incompressibility estimate ii. for general $F \in {\mathcal {C}}$ follows by a monotone class argument. The energy estimate iii. for general $F \in {\mathcal {C}}$ is slightly more subtle. We let $F_N = \mathbb {E}_\mu [F|{\mathcal {F}}_N]$ with $F_N = \Pi _N F_N$ and apply the incompressibility condition ii. to obtain

$$\begin{aligned} \mathbb {E}\left[ \sup _{t \leqslant T}\left| \int _0^t F(u_s) \textrm{d}s\right| \right]&= \lim _{N\rightarrow \infty } \mathbb {E}\left[ \sup _{t \leqslant T}\left| \int _0^t F_N(u_s) \textrm{d}s\right| \right] \\&\lesssim \limsup _{N\rightarrow \infty } (T^{1/2}+T) \Vert F_N\Vert _{{\mathcal {H}}^{-1}_0}. \end{aligned}$$

Now for $$G \in {\mathcal {C}}$$:

$$ \mathbb {E}[F_N G]=\mathbb {E}[F\mathbb {E}[G|{\mathcal {F}}_N]] \leqslant \Vert F\Vert _{{\mathcal {H}}^{-1}_0} \Vert \mathbb {E}[G|{\mathcal {F}}_N]\Vert _{{\mathcal {H}}^1_0}, $$

so the energy estimate follows if we can show that $G\mapsto \mathbb {E}[G|{\mathcal {F}}_N]$ is bounded linear map on ${\mathcal {H}}^1_0$. Since it is obviously bounded $L^2(\mu )$, it suffices to show boundedness in $\dot{{\mathcal {H}}}^1_0$:

$$\begin{aligned} \Vert \mathbb {E}[G|{\mathcal {F}}_N]\Vert _{\dot{{\mathcal {H}}}^1_0}^2&\simeq \sum _k \lambda _k \mathbb {E}[|D_{e_k}\mathbb {E}[G|{\mathcal {F}}_N]|^2] = \sum _{k\leqslant N} \lambda _k \mathbb {E}[|\mathbb {E}[D_{e_k}G|{\mathcal {F}}_N]|^2]\\&\leqslant \sum _k \lambda _k {\mathbb {E}}[|D_{e_k}G|^2] \simeq \Vert G\Vert _{\dot{{\mathcal {H}}}^1_0}^2. \end{aligned}$$

This proves the energy estimate for general $F \in {\mathcal {C}}$.

It remains to show that any limit point satisfies the weak solution property (iv.). Let $f \in S$, and note that

$$ u^N_t(f) = u^N_0(f) - \int _0^t u^N_s(Af)\textrm{d}s + \int _0^t \langle {:}B^N(u^N_s,u^N_s){:}, f\rangle _{V^*, V}\textrm{d}s + M^{f,N}_t. $$

The martingale and the initial condition converge to the desired limit by the usual arguments, see e.g. [17, 18]. For the linear drift there is a small problem because we do not know if $Af \in S$, and therefore we cannot use the the weak convergence in $C(\mathbb {R}_+, \mathbb {R})$ to describe the limit of $\int _0^\cdot u^N_s(Af)\textrm{d}s$. But by density of *S* in *H* and by the uniform incompressibility estimate we can approximate $\int _0^\cdot u^N_s(Af)\textrm{d}s$ by $\int _0^\cdot u^N_s(g)\textrm{d}s$ with $g \in S$ up to making an $L^1$-error of at most $\varepsilon$, pass to the limit $\int _0^\cdot u_s(g)\textrm{d}s$, and replace this by $\int _0^\cdot u_s(Af)\textrm{d}s$, again making an $L^1$-error of at most $\varepsilon$.

We are left with deriving the limit of the quadratic drift, and with showing that it has the required approximation property in *p*-variation. Let $\mathcal {B}^f$ be a limit point of $\mathcal {B}^{f,N} = \int _0^{\cdot } \langle {:} B^N (u^N_s, u^N_s) {:}, f \rangle _{V^{*}, V} \textrm{d}s$, which exists by tightness of this sequence, let $p' < 2$ be such that $( \Vert \mathcal {B}^{f,N} \Vert _{[0, T], p' - \operatorname {var}})_N$ is tight (see Lemma 3.4), and let $p \in (p', 2)$. By density of $\bigcup _K \Pi _K \mathcal {C}$, we can find for each $M \in \mathbb {N}$ a cylinder function $F_{M, f} = \Pi _{K_M} F_{M, f}$ with $\Vert \langle {:} \tilde{B}^{\rho _M} (\cdot , \cdot ) {:}, f \rangle _{V^{*}, V} - F_{M, f} \Vert _{L^2 (\mu )} \leqslant \frac{1}{M}$, and as $\left\| \int _0^{\cdot } (\cdot ) \textrm{d}s \right\| _{[0, T], p - \textrm{var}} \leqslant \int _0^T | (\cdot ) | \textrm{d}s$, this yields

$$\begin{aligned}&\mathbb {E} \left[ \left\| \mathcal {B}^f - \int _0^\cdot \langle {:} \tilde{B}^{\rho _M} (u_s, u_s){:}, f \rangle _{V^{*}, V} \textrm{d}s \right\| _{[0, T], p - \textrm{var}} \right] \\&\quad \leqslant \mathbb {E} \left[ \left\| \mathcal {B}^f - \int _0^{\cdot } F_{M, f} (u_s) \textrm{d}s \right\| _{[0, T], p - \textrm{var}} \right] + \frac{T}{M} . \end{aligned}$$

By an interpolation argument, tightness in *p*-variation uniform topology is equivalent to tightness in the uniform topology together with tightness of the $p'$-variation norm, see [15, Thm. 6.1]. Together with the convergence $\int _0^{\cdot } F_{M, f} (u_s^N) \textrm{d}s \rightarrow \int _0^{\cdot } F_{M, f} (u_s) \textrm{d}s$ in uniform 1-variation topology, we thus obtain with Fatou’s lemma for some sufficiently large $p<2$ (whose value will be fixed at the end of the proof),

66 $$\begin{aligned}&\mathbb {E} \left[ \left\| \mathcal {B}^f - \int _0^{\cdot } F_{M, f} (u_s) \textrm{d}s \right\| _{[0, T], p - \textrm{var}} \right] \nonumber \\&\leqslant \liminf _{N \rightarrow \infty } \mathbb {E} \left[ \left\| \int _0^{\cdot } \langle {:} B^N (u^N_s, u^N_s) {:}, f \rangle _{V^{*}, V} \textrm{d}s - \int _0^{\cdot } F_{M, f} (u_s^N) \textrm{d}s \right\| _{[0, T], p - \textrm{var}} \right] \nonumber \\&\lesssim \liminf _{N \rightarrow \infty } \mathbb {E}_{\mu ^N} \left[ \left\| \int _0^{\cdot } \langle {:} B^N (u^N_s, u^N_s) {:}, f \rangle _{V^{*}, V} \textrm{d}s\right. \right. \nonumber \\&\quad \left. \left. - \int _0^{\cdot } \langle {:} \tilde{B}^{\rho _M} (u^N_s, u^N_s) {:}, f \rangle _{V^{*}, V} \textrm{d}s \right\| _{[0, T], p - \textrm{var}}^2 \right] ^{1/2} + \frac{T}{M} \nonumber \\&\lesssim \varepsilon _M^{\beta } + \frac{T}{M} \end{aligned}$$

for some $\beta > 0$, where the last step will be justified in the remainder of this proof. Once (66) is established, we have shown that $\mathbb {E} [ \Vert \mathcal {B}^f - \int _0^\cdot \langle {:} \tilde{B}^{\rho _M} (u_s, u_s){:}, f \rangle _{V^{*}, V} \textrm{d}s\Vert _{[0, T], p - \textrm{var}} ]$ converges to 0 as $M\rightarrow \infty$ and this concludes the proof. To show (66) we use that, since $u^N = \Pi _N u^N$,

67 $$\begin{aligned}&\langle {:}B^N(u^N_s,u^N_s){:}, f\rangle _{V^*, V} - \langle {:}{\tilde{B}}^{\rho _M}(u^N_s,u^N_s){:}, f\rangle _{V^*, V}\nonumber \\&\quad = \langle {:}B(\Pi _N u^N_s,\Pi _N u^N_s){:}, \Pi _N f - f\rangle _{V^*, V} \nonumber \\&\qquad + \langle {:}B(\Pi _N u^N_s,\Pi _N u^N_s){:} -{:}{\tilde{B}}^{\rho _M}(\Pi _N u^N_s,\Pi _N u^N_s){:}, f\rangle _{V^*, V}. \end{aligned}$$

For the first term on the right hand side we simply bound with the Cauchy-Schwarz inequality

$$\begin{aligned}&\mathbb {E}_{\mu ^N}\left[ \left| \int _s^t \langle {:}B(\Pi _N u^N_r,\Pi _N u^N_r){:}, \Pi _N f - f\rangle _{V^*, V} \textrm{d}r\right| ^2\right] \\&\quad \lesssim (t-s)^2 \Vert \Pi _N\Vert _{L(H, V)} \Vert \Pi _N\Vert _{L(H, H)} \Vert \Pi _N f - f\Vert _{\dot{V}}\\&\quad \lesssim (t-s)^2 (1+\lambda _N)^{1/2}\lambda _N^{-1/2} \Vert (1-\Pi _N)Af\Vert _H \\&\quad \lesssim (t-s)^2 \Vert (1-\Pi _N)Af\Vert _H, \end{aligned}$$

where we used that

$$\begin{aligned} \Vert \Pi _N h \Vert _{V}^2 \simeq \sum _{k \leqslant N} (1+ \lambda _k) \langle h, e_k\rangle _H^2 \leqslant (1+\lambda _N) \Vert h\Vert _H^2 \end{aligned}$$

and

$$ \Vert \Pi _N f - f\Vert _{\dot{V}}^2 \simeq \sum _{k>N} \lambda _k \langle f, e_k\rangle _H^2 \leqslant \lambda _N^{-1}\sum _{k>N} \lambda _k^2 \langle f, e_k\rangle _H^2 = \lambda _N^{-1} \Vert (1 - \Pi _N) Af\Vert _H^2. $$

By the Besov-variation embedding already used in Lemma 2.20, we get for any $p<2$

68 $$\begin{aligned} \lim _{N \rightarrow \infty } \mathbb {E}_{\mu ^N} \left[ \left\| \int _0^{\cdot } \langle {:}B(\Pi _N u^N_s,\Pi _N u^N_s){:}, \Pi _N f - f\rangle _{V^*, V} \textrm{d}s \right\| _{[0, T], p - \textrm{var}}^2 \right] ^{1/2} = 0. \end{aligned}$$

For the remaining term in (67) we use the same interpolation argument as in the proof of Lemma 2.20 together with the bound $\Vert \Pi _N^{\otimes 2}\Vert _{L(V\otimes _s H, V\otimes _s H)} \lesssim 1$ from Lemma A.3 in the appendix, to obtain a bound on the increment:

69 $$\begin{aligned} \mathbb {E}_{\mu ^N}\left[ \left| \int _s^t \langle {:}B(\Pi _N u^N_r,\Pi _N u^N_r){:} -{:}{\tilde{B}}^{\rho _M}(\Pi _N u^N_r,\Pi _N u^N_r){:}, f\rangle _{V^*, V} \textrm{d}r\right| ^2\right] \lesssim \varepsilon _M^\beta (t-s)^{1+\zeta } \end{aligned}$$

for some $\beta ,\zeta >0$. If $p<2$ is such that $2/p < 1+\zeta$ and such that there exists $p'\in (p,2)$ for which $( \Vert \mathcal {B}^{f,N} \Vert _{[0, T], p' - \operatorname {var}})_N$ is tight, we combine (67), (68), (69) and we use the same Besov-variation embedding as in Lemma 2.20 to deduce (66). $\square$

### Uniqueness proof: Duality of semigroup and energy solutions

To prove the uniqueness of energy solutions, we will show that every energy solution solves the martingale problem for the operator $({\mathcal {D}}_{\textrm{max}}, {\mathcal {L}})$ constructed in Proposition 3.1 and Lemma 3.2. For that purpose, we first extend the weak solution property from linear test functions to cylinder functions.

#### Lemma 3.6

(Itô formula) Let *u* be an energy solution and let $F \in \mathcal {C}$. Then

$$\begin{aligned} F(u_t) - F(u_0) - I (\mathcal {L}F)_t, \qquad t \geqslant 0, \end{aligned}$$

is a continuous martingale.

#### Proof

This follows by the same arguments as [21, Lem. 4.14], relying on Itô’s formula, Young integration and the convergence of $\int _0^\cdot \langle {:} {\tilde{B}}^{\rho _N} (u_s, u_s) {:}, f \rangle _{V^{*}, V} \textrm{d}s$ to $I (\langle {:} B (\cdot , \cdot ) {:}, f \rangle _{V^{*}, V})$ in *p*-variation. $\square$

#### Theorem 3.7

(Uniqueness of energy solutions) Let $\nu \ll \mu$ with $\frac{\textrm{d}\nu }{\textrm{d}\mu } \in L^2 (\mu )$ and let $(u_t)_{t \geqslant 0}$ be an energy solution with $u_0 \sim \nu$. Then *u* is a solution to the martingale problem for $(\mathcal {D}_{\max }, \mathcal {L})$, i.e. for any $F \in \mathcal {D}_{\max }$ it holds that

$$ F (u_t) - F (u_0) - \int _0^t \mathcal {L} F (u_s) \textrm{d}s, \qquad t \geqslant 0, $$

is a martingale in the filtration generated by *u*. Consequently, the law of *u* is uniquely determined by its initial condition, it is a Markov process and it has $\mu$ as an invariant measure.

#### Proof

To see that *u* is a solution to the martingale problem for $(\mathcal {D}_{\max }, \mathcal {L})$, let $F \in \mathcal {D}_{\max }$. By Lemma 3.2, there exists $F^{\sharp } \in L^2 (\mu )$ with $F =\mathcal {R}_1 F^{\sharp }$, and again by Lemma 3.2 the set $(1 -\mathcal {L}) \mathcal {C}$ is dense in $\mathcal {H}^{- 1}_0$. Therefore, there exists a sequence $(F^N)_N \subset \mathcal {C}$ with $(1 -\mathcal {L}) F^N \rightarrow F^{\sharp }$ in $\mathcal {H}^{- 1}_0$. By Proposition 3.1 we have $\mathcal {R}_1 \in L (\mathcal {H}^{- 1}_0, \mathcal {H}^1_0)$, and therefore the following convergence holds in $\mathcal {H}^1_0$:

$$ F^N =\mathcal {R}_1 (1 -\mathcal {L}) F^N \rightarrow \mathcal {R}_1 F^{\sharp } = F, $$

where the first equality follows from the uniqueness of solutions to the resolvent equation in Lemma 3.2, as

$$ (1 -\mathcal {L}) F^N = (1 -\mathcal {L}) (\mathcal {R}_1 (1 -\mathcal {L}) F^N) . $$

We now use this convergence to justify that *u* solves the martingale problem for the test function *F*. By the Itô formula in Lemma 3.6, the process

$$\begin{aligned} F^N (u_t) - F^N (u_0) - I (\mathcal {L}F^N)_t, \qquad t \geqslant 0, \end{aligned}$$

is a continuous martingale, and we will pass to the limit $N \rightarrow \infty$ in each term. First observe that $F^N (u_t) - F^N (u_0) \rightarrow F (u_t) - F (u_0)$ in $L^1 (\mathbb {P})$, since by the uniform $L^2$ bound for the density ii.,

$$ |\mathbb {E}[(F - F^N) (u_t) - (F - F^N) (u_0)] | \lesssim \Vert F - F^N \Vert _{L^2 (\mu )} \lesssim \Vert F - F^N \Vert _{\mathcal {H}^1_0} \rightarrow 0. $$

For the integral term, we use that $(1 -\mathcal {L}) F = F^{\sharp }$ and combine this with the energy estimate iii. to obtain

$$\begin{aligned} \mathbb {E} [|I (\mathcal {L}(F - F^N))_t |]&=\mathbb {E} [|I ((F - F^N) - (F^{\sharp } - (1 -\mathcal {L}) F^N))_t |]\\&\lesssim \Vert (F - F^N) - (F^{\sharp } - (1 -\mathcal {L}) F^N)\Vert _{\mathcal {H}^{- 1}_0} \rightarrow 0. \end{aligned}$$

The martingale property is preserved under $L^1$-limits and thus *u* is a solution to the martingale problem for $(\mathcal {D}_{\max }, \mathcal {L})$.

Now uniqueness of *u*, Markov property and invariance of $\mu$ follow by the same arguments as in [21, Theorem 4.8]: The martingale problem extends to time-dependent test functions $F \in C^1_T L^2 (\mu ) \cap C_T \mathcal {D}_{\max }$ by writing $F (t, u_t) - F (0, u_0) = \sum _{k = 0}^{n - 1} (F (t_{k + 1}^n, u_{t^n_{k + 1}}) - F (t^n_k, u_{t^n_k}))$ for $t^n_k = k t / n$, and using a Taylor expansion of *F* in *t*, and the martingale problem for time-independent test functions in the *u* variable. This shows that

$$ F (t, u_t) - F (0, u_0) - \int _0^t (\partial _s +\mathcal {L}) F (s, u_s) \textrm{d}s, \qquad t \in [0, T], $$

is a continuous martingale, and in particular it has vanishing expectation. Now it suffices to take $F (t, u) = P_{T - t} G (u)$ for $G \in \mathcal {D}_{\max }$ and $(P_t)_{t \geqslant 0}$ the semigroup from Proposition 3.1, which leads to

$$\begin{aligned} \mathbb {E} [G (u_T)] =\mathbb {E} [P_T G (u_0)], \end{aligned}$$

so we obtain the uniqueness of one-dimensional marginals. The usual iteration as in [12, Theorem 4.4.2] or [21, Theorem 4.8] then yields uniqueness of the finite-dimensional distributions. $\square$

#### Remark 3.8

(Ergodicity of energy solutions) Assume $v = 0$ is the only element of *V* with $\Vert v \Vert _{\dot{V}} = 0$. Then the invariant measure $\mu$ is ergodic for the Markov process *u* from the previous theorem. Indeed, it suffices to show that if $F \in \mathcal {D}_{\max }$ solves $\mathcal {L}F = 0$, then *F* is a.s. constant. But we know from Lemma 3.2 that

$$ 0 = \langle -\mathcal {L}F, F \rangle _{\mathcal {H}^{- 1}_0, \mathcal {H}^1_0} = \langle -\mathcal {L}_0 F, F \rangle _{\mathcal {H}^{- 1}_0, \mathcal {H}^1_0} \simeq \mathbb {E} [\Vert D F \Vert _{\dot{V}}^2], $$

so by assumption $D F = 0$ a.s. and thus *F* is a.s. constant.

## Examples

Here we discuss in detail the examples mentioned in the introduction. In each example we identify $H,V,A,B,(\rho _N)$ and verify the conditions in Assumptions 1 and 1. For Assumption 1 we do not explicitly verify that $\Vert B (v, w) \Vert _{\dot{V}^{*}} \lesssim \Vert v\Vert _V \Vert w\Vert _H + \Vert v\Vert _H \Vert w\Vert _V$, and instead we only show the stronger condition $\Vert B (\varphi ) \Vert _{\dot{V}^{*}} \lesssim \Vert \varphi \Vert _{V \otimes _s H}$, which controls also $B(v,w) = B (v \otimes w)$.

### Example 4.1

(Multi-component Burgers equation with Dirichlet boundary conditions on (0, 1) or $(0, \infty ))$ Consider the multi-component stochastic Burgers equation on $D \in \{ (0, 1), (0, \infty ) \}$ with homogeneous Dirichlet boundary conditions: Formally, $u: \mathbb {R}_+ \times D \rightarrow \mathbb {R}^d$ with

$$ \partial _t u^i = \Delta u^i + {: }\sum _{j, k} \Gamma ^i_{j k} \partial _x (u^j u^k) {:} + \sqrt{2 (- \Delta )} \xi ^i, $$

where $\Gamma$ is symmetric in its 3 arguments. We choose $H = L^2 (D \times \{ 1, \ldots , d \}) = L^2 (D)^d$, the smooth functions are $S = C^{\infty }_c (D \times \{ 1, \ldots , d \}) = C^{\infty }_c (D)^d$, and $V = H^1_0 (D \times \{ 1, \ldots , d \}) = H^1_0 (D)^d$ with norm

$$ \Vert v \Vert _V^2 = \sum _j (\Vert v^j \Vert _{L^2}^2 + \Vert \partial _x v^j \Vert _{L^2}^2), $$

and $$\Vert v \Vert _{\dot{V}}^2 = \Vert v \Vert _V^2 - \Vert v \Vert _H^2 = \sum _j \Vert \partial _x v^j \Vert _{L^2}^2$$.

We define approximations similarly to [25] by averaging over a rescaled indicator function with ”slowed" shifting near the boundary in order to stay inside of the domain. More precisely, set $x_{\text {max}}=\sup _{y\in D}y\in \{1,\infty \}$, and with interpretation $\infty +r=\infty$, $r\in \mathbb {R}$, we let $p_N: D \rightarrow (0,x_{\text {max}}-1/N)$ be a $C^1$, monotone function such that $p_N(x) = x$ for $x \in (0,\,x_{\text {max}}-2/N)$. We additionally assume $p_N$ satisfies

70 $$\begin{aligned} 0 < \inf _{x,N} p_N'(x) \leqslant \sup _{x,N} p_N'(x) = 1. \end{aligned}$$

We define

$$ \rho _N h (x) = \int _D N \boldsymbol{1}_{I^N_x} (y) h (y) \textrm{d}y,\qquad \text {for} \qquad I_x^N = (p_N(x), p_N(x) + 1 / N) , $$

which can be written as

71 $$\begin{aligned} \rho _N h=(\iota _N*h)\circ p_N,\qquad \text {for} \qquad \iota _N=N 1_{(-1,0)}(N\cdot ). \end{aligned}$$

We will also consider the Sobolev norms

$$ \Vert v \Vert _{H^{\alpha }}^2 = \left\{ \begin{array}{ll} \Vert v \Vert _{L^2}^2, &  \alpha = 0,\\ \Vert v \Vert _{L^2}^2 + \int _{D^2} \frac{| v (x) - v (y) |^2}{| x - y |^{1 + \alpha }} \textrm{d}x \textrm{d}y, &  \alpha \in (0, 1),\\ \Vert v \Vert _{L^2}^2 + \Vert \partial _x v \Vert _{L^2}^2, &  \alpha = 1, \end{array}\right. $$

extended to vector-valued functions *v* as above, and $H^\alpha _0$ is the closure of the smooth compactly supported functions in $H^\alpha$. The homogeneous norms $\Vert v \Vert _{\dot{H}^{\alpha }}^2$ are defined by omitting the squared $L^2$ norm on the right hand side.

Let us verify the assumptions on $A = - \Delta$: For $f, g \in S$ we obtain, using that $f^j, g^j$ are compactly supported in *D* to justify the integration by parts,

$$ \langle A f, g \rangle _{V^{*}, V} = \sum _j \langle \partial _x f^j, \partial _x g^j \rangle _{L^2} = \langle f, A g \rangle _{V^{*}, V}, $$

and thus

$$\begin{aligned} \langle A f, g \rangle _{V^{*}, V}&= \sum _j \langle \partial _x f^j, \partial _x g^j \rangle _{L^2} \leqslant \left( \sum _j \Vert \partial _x f^j \Vert ^2_{L^2} \right) ^{1 / 2} \left( \sum _j \Vert \partial _x f^j \Vert ^2_{L^2} \right) ^{1 / 2}\\  &\simeq \Vert f \Vert _{\dot{V}} \Vert g \Vert _{\dot{V}}, \end{aligned}$$

and similarly

$$ \langle A f, f \rangle _{V^{*}, V} = \sum _j \Vert \partial _x f^j \Vert ^2_{L^2} = \Vert f \Vert _{\dot{V}}^2 . $$

To verify the assumptions on $B (f, g) = \sum _{j, k} \Gamma ^i_{j k} \partial _x (f^j g^k)$, we first observe that $V \otimes _s H \subset H \otimes _s H \simeq L^2_s ((D \times \{ 1, \ldots , d \})^2)$, where the subscript *s* means that we consider functions that are symmetric in the sense that $\varphi ^{i_1, i_2} (x_1, x_2) = \varphi ^{i_2, i_1} (x_2, x_1)$, and

$$\begin{aligned} \Vert \varphi \Vert _{V \otimes _s H}^2&= \!\sum _{i_1, i_2} \int _{D^2}\! \left( | \varphi ^{i_1, i_2} (x, y) |^2 \!+ \frac{1}{2} (| \partial _x \varphi ^{i_1, i_2} (x, y) |^2 \!+ | \partial _y \varphi ^{i_1, i_2} (x, y) |^2) \right) \textrm{d}x \textrm{d}y \\&\simeq \Vert \varphi \Vert _{H^1_0 (D^2)^{d^2}}^2, \end{aligned}$$

so that we can identify $V \otimes _s H$ with a symmetric subspace of $H^1_0 (D^2)^{d^2}$. With the trace operator $T\varphi (x) = \varphi (x,x)$ we obtain for smooth and compactly supported $f, \varphi$ and $\alpha \in [0,1]$

72 $$\begin{aligned} \langle B (\varphi ), f \rangle _{V^{*}, V}&= \sum _{i, j, k} \Gamma ^i_{j k} \int _D \partial _x \varphi ^{j, k} (x, x) f^i (x) \textrm{d}x \lesssim \sum _{i, j, k} \Vert \partial _x T \varphi ^{j,k}\Vert _{(\dot{H}^\alpha _0)^*} \Vert f_i\Vert _{\dot{H}^\alpha }. \end{aligned}$$

By integration by parts we see that the derivative $\partial _x$ is in $L(\dot{H}^1,L^2)\cap L(L^2,(\dot{H}^1_0)^*)$, so by interpolation it is also in $L(H^{1-\alpha },(\dot{H}^{\alpha }_0)^*)$. Moreover, by Lemma A.7 the trace operator is continuous from $H^{\beta } (D^2)$ to $H^{\beta - \frac{1}{2}} (D)$. Therefore, we can bound for $\alpha <1$

73 $$\begin{aligned} \Vert \partial _x T \varphi \Vert _{(\dot{H}^\alpha _0)^*} \lesssim \Vert T\varphi \Vert _{H^{1-\alpha }} \lesssim \Vert \varphi \Vert _{H^{3/2 - \alpha }(D^2)}. \end{aligned}$$

For $\alpha =1/2$ we obtain the required estimate for $B:V\otimes _s H \rightarrow \dot{V}^*$. Moreover, using the symmetry of $\Gamma$ in its arguments *i*, *j*, *k* and integration by parts, we see that for $f \in S$

$$ \langle B (f, f), f \rangle _{V^{*}, V} = \sum _{i, j, k} \Gamma ^i_{j k} \int _D \partial _x (f^j (x) f^k (x)) f^i (x) \textrm{d}x = 0, \qquad f \in S. $$

Our remaining task is to verify that the approximation $(\rho _N)$ has the required properties. We know from Lemma A.5 that $\sup _N \Vert \rho _N \Vert _{L (H, H)} + \Vert \rho _N \Vert _{L (\dot{V}, \dot{V})} < \infty$ and $\Vert \rho _N\Vert _{L(H,V)} \lesssim N$, and for $f \in S$ and $\varphi \in {\mathcal {C}}^\infty _c(D^2)$ we obtain

$$\begin{aligned} \Vert \rho _N f - f \Vert _V&\lesssim N^{-1} \Vert f\Vert _{H^2}, \end{aligned}$$

as well as by (72) with $\alpha =0$, Lemma A.5 and (73),

$$\begin{aligned} \langle B(\varphi ),(\rho _N-\text {Id})f\rangle _{V^*,V} \lesssim \Vert \varphi \Vert _{H^{3/2}(D^2)}\Vert (\rho _N-\text {Id})f\Vert _{L^2}\lesssim N^{-1}\Vert \varphi \Vert _{H^{3/2}(D^2)}\Vert f\Vert _{H^1} , \end{aligned}$$

and also for any $\kappa \in (0,\tfrac{1}{2})$,

74 $$\begin{aligned} | \langle B(\rho _N^{\otimes 2} \varphi -\varphi ), f \rangle _{V^{*}, V} |&\lesssim \Vert \rho _N^{\otimes 2} \varphi -\varphi \Vert _{H^{1/2+\kappa }(D^2)} \Vert f\Vert _{\dot{H}^{1-\kappa }}\nonumber \\  &\lesssim N^{\kappa -1/2}\Vert \varphi \Vert _{H^1(D^2)}\Vert f\Vert _{H^{1}}, \end{aligned}$$

where the last step follows by another application of Lemma A.5 together with a tensorization argument. Therefore, Assumptions 1 and 1 are satisfied in this example. The conditions for our existence theorem are satisfied if $D=(0,1)$ (with $e_k(x) = \sqrt{2} \sin (k\pi x)$), but not for $D=(0,\infty )$.

### Remark 4.2

Although uniqueness holds on non-compact domains such as $(0,\infty )$ (since it only relies on Assumptions (A) and (B)), the existence result of Theorem 3.5 does not apply in that setting because here we assume that *A* admits an orthonormal basis of eigenvectors, which fails for the Laplacian on $(0,\infty )$. See Remark 1.7 for a possible strategy that could prove the existence of energy solutions also in that case.

To simplify the presentation we restrict to scalar-valued equations from now on, but in all of the following examples it would also be possible to consider systems of equations.

### Example 4.3

(Burgers equation with elliptic differential operator and Dirichlet Boundary conditions) We consider the same equation as before, again on a domain $D \in \{ (0, 1), (0, \infty ) \}$, and for simplicity we assume $d = 1$ (scalar-valued equation). We change the operator to

$$\begin{aligned} A v = - \partial _x (a \partial _x v), \end{aligned}$$

for a measurable function *a* such that there exists $\varepsilon > 0$ with $a (x) \in [\varepsilon , \varepsilon ^{- 1}]$ for all $x \in D$. The spaces $H, S, V, \dot{V}$ have the same definition as before, as does the approximation $(\rho _N)$. Since $f, g \in S$ have compact support, we obtain

$$\begin{aligned} \langle A f, g \rangle _{V^*, V} = - \int _D \partial _x (a \partial _x f) (x) g (x) \textrm{d}x = \int _D a(x) \partial _x f (x) \partial _x g (x) \textrm{d}x, \end{aligned}$$

and thus

$$\begin{aligned}\langle A f, g \rangle _{V^{*}, V} \leqslant \varepsilon ^{-1} \Vert \partial _x f \Vert _{L^2} \Vert \partial _x g \Vert _{L^2} \lesssim \Vert f \Vert _{\dot{V}} \Vert g \Vert _{\dot{V}}, \quad \langle A f, f \rangle _{V^{*}, V} \geqslant \varepsilon \Vert \partial _x f \Vert ^2_{L^2} = \varepsilon \Vert f \Vert _{\dot{V}}^2 . \end{aligned}$$

### Example 4.4

(Hyperviscous Burgers equation with Neumann boundary conditions on (0, 1)) Let $\theta \in (1, 2]$. We consider the equation (for simplicity scalar-valued)

$$ \partial _t u = - (1 - \Delta )^{\theta } u + {:} \left( \partial _x u^2 - \frac{2}{3} u^2 (\delta _1 - \delta _0) \right) {:} + \sqrt{2 (1 - \Delta )^{\theta }} \xi $$

on (0, 1), where $\Delta$ is the Laplacian with homogeneous Neumann boundary conditions and we define $(1 - \Delta )^{\theta }$ using functional calculus, which can be made explicit with the eigenbasis $(\cos (\pi k \cdot ))_{k \in \mathbb {N}_0}$ of $\Delta$. The term $- \frac{2}{3} u^2 (\delta _1 - \delta _0)$ represents a boundary renormalization that is necessary to guarantee the property $\langle B (f, f), f \rangle _{V^{*}, V} = 0$, and for $\theta = 1$ (which we exclude!) we could formally interpret it as a change of homogeneous Neumann boundary conditions to nonlinear Robin type boundary conditions $u' (1) = \frac{2}{3} u^2 (1)$ and $u' (0) = \frac{2}{3} u^2 (0)$, see [25] for a detailed discussion in a related context. We take $H = L^2 ((0, 1))$, the smooth functions are $S = \{ f \in C^{\infty } ([0, 1]): f' (0) = f' (1) = 0 \}$, and *V* is the closure of *S* in $H^{\theta } ((0, 1))$, where with the orthonormal basis of eigenfunctions $(e_k)_{k \in \mathbb {N}_0}$ of $(1-\Delta )$ given by $e_k = \sqrt{2} \cos (k\pi x)$ for $k>0$ and $e_0 = 1$,

$$ \Vert f\Vert _{H^\alpha }^2 := 2 \sum _{k=0}^\infty (1+k^2)^{\alpha } \langle f, e_k\rangle _H^2, $$

and $H^\alpha$ consists of those functions in $L^2$ for which the norm is finite. Due to the factor 2 in front of the sum and since $(e_k)$ is an orthonormal basis in *H*, we have $\dot{V} = V$.

We use the spectral approximation

$$ \rho _N h(x) = \sum _{k=0}^N e_k(x) \langle h, e_k \rangle _H. $$

The estimates for *A* follow by a simple explicit computation using the orthonormal basis $(e_k)$, so let us focus on the bound for *B*: We can identify $V \otimes _s H$ with a subspace of $H^{\theta } ((0, 1)^2)$, and for smooth $\varphi$ in this space and for $f \in S$ we have with $T \varphi (x) = \varphi (x, x)$ for any $\alpha \in [0,1)$ and any $\kappa >0$

$$\begin{aligned} \langle B (\varphi ), f \rangle _{V^{*}, V}&= \int _0^1 \partial _x \varphi (x, x) f (x) \textrm{d}x - \frac{2}{3} (\varphi (1, 1) f (1) - \varphi (0, 0) f (0))\\&\lesssim \Vert \partial _x T \varphi \Vert _{H^{-\alpha }} \Vert f \Vert _{H^{\alpha }} + \Vert \varphi \Vert _{\infty } \Vert f \Vert _{\infty } \lesssim \Vert T \varphi \Vert _{H^{3/2+\kappa -\alpha }} \Vert f \Vert _{H^{\alpha }}\\&\quad + \Vert \varphi \Vert _{\infty } \Vert f \Vert _{\infty } \\&\lesssim \Vert \varphi \Vert _{H^{2+\kappa -\alpha }} \Vert f \Vert _{H^{\alpha }} + \Vert \varphi \Vert _{\infty } \Vert f \Vert _{\infty }, \end{aligned}$$

where we applied Lemma A.8 from the Appendix to bound $\partial _x$ (here we need $\alpha <1+\kappa$) and we used that $T: H^{2+\kappa -\alpha } \rightarrow H^{3/2+\kappa -\alpha }$ as $3/2+\kappa -\alpha > 0$ (see the Fourier based computation in Lemma A.7 which works similarly for the Neumann Sobolev spaces). Moreover, for any $\beta >1$ we can bound $\Vert \varphi \Vert _\infty \lesssim \Vert \varphi \Vert _{H^\beta }$ and $\Vert f\Vert _\infty \lesssim \Vert f\Vert _{H^{\beta /2}}$, again by a spectral computation. Therefore, using that $\theta >1$, we can find a small $\varepsilon >0$ such that

$$ \langle B (\varphi ), f \rangle _{V^{*}, V}\lesssim \Vert \varphi \Vert _{H^{\theta -\varepsilon }} \Vert f \Vert _{H^{\theta -\varepsilon }}. $$

Moreover, due to the boundary renormalization we obtain for all $f \in S$

$$ \langle B (f, f), f \rangle _{V^{*}, V} = \int _0^1 \frac{2}{3} \partial _x f^3 (x) \textrm{d}x - \frac{2}{3} (f (1)^3 - f (0)^3) = 0. $$

Another simple Fourier computation shows that $\rho _N$ satisfies the same bounds as in Lemma A.5, actually for all $0\leqslant \alpha \leqslant \beta$, without requiring $\beta \leqslant 1$. Therefore, the bounds $\Vert \rho _N f - f\Vert _{V} \lesssim N^{-\theta } \Vert f\Vert _{H^{2\theta }}$ and

$$ |\langle B (\rho _N^{\otimes 2}\varphi ), f \rangle _{V^{*}, V} - \langle B (\varphi ), f \rangle _{V^{*}, V}| \lesssim N^{-\varepsilon } \Vert \varphi \Vert _{V\otimes _s H} \Vert f\Vert _{\dot{V}} $$

follow as in Example 4.1.

We have shown that Assumptions 1 and 1 are satisfied in this example, and we already heavily used that *A* can be diagonalized, so that we also obtain the existence of energy solutions. Unfortunately we required $\theta > 1$ both for estimating the integral part and the boundary part of *B*, and thus we are unable to solve the non-fractional stochastic Burgers equation with (renormalized) homogeneous Neumann boundary conditions.

### Example 4.5

(Stochastic Burgers equation with regional fractional Laplacian) We consider the domain $D = (0, 1)$ and $\gamma \in \left[ \frac{3}{2}, 2 \right)$ and the equation studied in [2], formally $u: \mathbb {R}_+ \times D \rightarrow \mathbb {R}$,

$$ \partial _t u =\mathbb {L}^{\gamma / 2} u + \partial _x u^2 + \sqrt{2 (-\mathbb {L}^{\gamma / 2})} \xi , $$

where $\mathbb {L}^{\gamma / 2}$ is the regional fractional Laplacian, given for some fixed $c > 0$ by

$$ \mathbb {L}^{\gamma / 2} f (x) :=\lim _{\varepsilon \rightarrow 0} c \int _0^1 \boldsymbol{1}_{\{ | y - x | \geqslant \varepsilon \}} \frac{f (y) - f (x)}{| y - x |^{1 + \gamma }} \textrm{d}y, $$

which is well-defined and continuous as a uniform limit if

$$ f \in S :=\{ f \in C^{\infty } ([0, 1]) : f (0) = f' (0) = f (1) = f' (1) = 0 \} . $$

Let $H = L^2 (D)$ and *V* is the closure of *S* in the Sobolev-Slobodeckij/Besov space $H^{\gamma / 2} (D) = B^{\gamma / 2}_{2, 2} (D)$ that already appeared in Example 4.1.

As $\gamma / 2 < 1$, the constraint $f' (0) = f' (1) = 0$ on the derivative of $f \in S$ has no effect on the closure, which equals $H^{\gamma / 2}_0$. We have that $\Vert f \Vert _{\dot{V}}^2 = \int _0^1 \int _0^1 \frac{(f (y) - f (x))^2}{| y - x |^{1 + \gamma }} \textrm{d}x \textrm{d}y$. We use the same approximation $\rho _N$ as in Example 4.1.

For $f, g \in S$ we have the following integration by parts rule from [19, Thm. 3.3]:

$$\begin{aligned} \int _0^1 (-\mathbb {L}^{\gamma / 2}) f (x) g (x) \textrm{d}x = \frac{c}{2} \int _0^1 \int _0^1 \frac{(f (y) - f (x)) (g (y) - g (x))}{| y - x |^{1 + \gamma }} \textrm{d}x \textrm{d}y \lesssim \Vert f \Vert _{\dot{V}} \Vert g \Vert _{\dot{V}} . \end{aligned}$$

For $f = g$, we obtain

$$ \int _0^1 (-\mathbb {L}^{\gamma / 2}) f (x) f (x) \textrm{d}x \simeq \Vert f \Vert _{\dot{V}}^2 . $$

The estimates for *B* are similar as in the case of the (usual) Dirichlet Laplacian on (0, 1): We can again identify $V \otimes _s H$ with a subspace of $H^{\gamma / 2}_0 (D^2)$, and from (74) we obtain for any $\kappa \in (0,1/2)$ the bound on the approximation

75 $$\begin{aligned}&| \langle B(\rho _N^{\otimes 2}\varphi ), f \rangle _{V^{*}, V} - \langle B(\varphi ), f \rangle _{V^{*}, V} | \leqslant \Vert \rho _N^{\otimes 2} \varphi -\varphi \Vert _{H^{1/2+\kappa }(D^2)} \Vert f\Vert _{\dot{H}^{1-\kappa }}, \end{aligned}$$

and similarly without approximation

76 $$\begin{aligned} | \langle B(\varphi ), f \rangle _{V^{*}, V} | \leqslant \Vert \varphi \Vert _{H^{1/2+\kappa }(D^2)} \Vert f\Vert _{\dot{H}^{1-\kappa }}. \end{aligned}$$

This second estimate leads to the requirement $\tfrac{\gamma }{2} \leqslant \tfrac{3}{4}$ or $\gamma \leqslant \tfrac{3}{2}$, because if $\gamma$ is smaller than that we cannot achieve $\tfrac{1}{2} + \kappa \leqslant \tfrac{\gamma }{2}$ and $1-\kappa \leqslant \tfrac{\gamma }{2}$ simultaneously. Note that $\gamma =\tfrac{3}{2}$ corresponds to the scaling critical case. To get a small factor from (75) we take $\kappa \in (0,\tfrac{1}{4})$ if $f \in S$, and we take $\kappa \in (\tfrac{1}{4},\tfrac{1}{2})$ if $\varphi \in H^{\gamma }_0(D^2) \subset S \otimes _s S$, and we apply Lemma A.5. The small factor for $\langle B(\varphi ), (\rho _N-\text {Id})\cdot \rangle _{V^{*}, V}$ follows similarly from (76) and Lemma A.5.

Finally, we obtain for $f \in S$

$$ \langle B (f, f), f \rangle _{V^{*}, V} = \int _D \partial _x (f (x) f (x)) f (x) \textrm{d}x = \frac{2}{3} \int _D \partial _x f^3 (x) \textrm{d}x = 0, \qquad v \in V. $$

Therefore, Assumptions 1 and 1 are satisfied. Also, *A* is diagonalizable because *V* is compactly embedded in *H*, and therefore our existence proof applies. But actually the existence of energy solutions for this example was previously shown by Cardoso and Gonçalves [2], who derive them as scaling limits of fluctuations in long range interacting particle systems. They consider only the stationary case, but the derivation should extend without problems to initial conditions with $L^2$ density or even $L^1$ density as in [23].

Apart from *A* being diagonalizable, everything should extend also to $(0,\infty )$ instead of (0, 1).

## Data Availability

No datasets were generated or analysed during the current study.

## Auxiliary computations

### Lemma A.1

The assumption $\langle B (v, v), v \rangle _{V^{*}, V} \equiv 0$ together with the symmetry and bilinearity of *B* implies

$$ \langle B (v_1, v_2), v_3 \rangle _{V^{*}, V} + \langle B (v_3, v_1), v_2 \rangle _{V^{*}, V} + \langle B (v_2, v_3), v_1 \rangle _{V^{*}, V} = 0 $$

for all $$v_1, v_2, v_3 \in V$$.

### Proof

By assumption we have for $v, w \in V$

$$\begin{aligned} 0&= \langle B (v + w, v + w), v + w \rangle _{V^{*}, V} + \langle B (v - w, v - w), v - w \rangle _{V^{*}, V}\\&= \langle B (v, v), w \rangle _{V^{*}, V} + 2 \langle B (v, w), v \rangle _{V^{*}, V} + 2 \langle B (v, w), w \rangle _{V^{*}, V} + \langle B (w, w), v \rangle _{V^{*}, V}\\&\quad - \langle B (v, v), w \rangle _{V^{*}, V} - 2 \langle B (v, w), v \rangle _{V^{*}, V} + 2 \langle B (v, w), w \rangle _{V^{*}, V} + \langle B (w, w), v \rangle _{V^{*}, V}\\&= 4 \langle B (v, w), w \rangle _{V^{*}, V} + 2 \langle B (w, w), v \rangle _{V^{*}, V}, \end{aligned}$$

so that the claim follows if $v_1 = v_2$, i.e.

$$ \langle B (w, w), v \rangle _{V^{*}, V} = - 2 \langle B (v, w), w \rangle _{V^{*}, V} . $$

Now we apply this identity repeatedly to obtain

$$\begin{aligned}&2 \langle B (v_1, v_2), v_3 \rangle _{V^{*}, V} + \langle B (v_1, v_1), v_3 \rangle _{V^{*}, V} + \langle B (v_2, v_2), v_3 \rangle _{V^{*}, V}\\&= \langle B (v_1 + v_2, v_1 + v_2), v_3 \rangle _{V^{*}, V}\\&= - 2 \langle B (v_1 + v_2, v_3), v_1 + v_2 \rangle _{V^{*}, V}\\&= - 2 \left( \langle B (v_1, v_3), v_1 \rangle _{V^{*}, V} + \langle B (v_2, v_3), v_2 \rangle _{V^{*}, V}\right. \\  &\quad \left. + \langle B (v_1, v_3), v_2 \rangle _{V^{*}, V} + \langle B (v_2, v_3), v_1 \rangle _{V^{*}, V} \right) \\&= \langle B (v_1, v_1), v_3 \rangle _{V^{*}, V} + \langle B (v_2, v_2), v_3 \rangle _{V^{*}, V} - 2 (\langle B (v_1, v_3), v_2 \rangle _{V^{*}, V}\\  &\quad + \langle B (v_2, v_3), v_1 \rangle _{V^{*}, V}), \end{aligned}$$

and subtracting $\langle B (v_1, v_1), v_3 \rangle _{V^{*}, V} + \langle B (v_2, v_2), v_3 \rangle _{V^{*}, V}$ on both sides and dividing by 2, we end up with

$$ \langle B (v_1, v_2), v_3 \rangle _{V^{*}, V} = - \langle B (v_1, v_3), v_2 \rangle _{V^{*}, V} - \langle B (v_2, v_3), v_1 \rangle _{V^{*}, V} . $$

$$\square $$

We would like to give an alternative proof of Lemma 2.15, without relying on the chaos expansion. This is based on the following lemma:

### Lemma A.2

$$ \mathcal {N}D_h = D_h (\mathcal {N}- 1) \boldsymbol{1}_{\mathcal {N} \geqslant 1}, \qquad \mathcal {N} \delta = \delta (\mathcal {N}+ 1). $$

### Proof

By the commutation relation of $D_h$ and $\delta$, see in [32, eq. (1.46)], we have

$$\begin{aligned} {}[\mathcal {N}, D_h] F&= \delta D D_h F - D_h \delta D F\\&= \delta D_h D F - D_h \delta D F\\&= D_h \delta D F - \langle D F, h \rangle _H - D_h \delta D F\\&= - D_h F. \end{aligned}$$

The second claim follows by duality of $\delta$ and *D*. $\square$

### Alternative proof of Lemma 2.15

Applying the previous result, we have

$$\begin{aligned} \mathbb {E} [\Vert D^2 F \Vert _{H \otimes _s \dot{V}}^2]&= \sum _{\ell _1, \ell _2} \mathbb {E} \left[ \left\langle D D_{e_{\ell _1}} F, D D_{e_{\ell _2}} F \right\rangle _H \right] \langle e_{\ell _1}, e_{\ell _2} \rangle _{\dot{V}}\\&= \sum _{\ell _1, \ell _2} \mathbb {E} \left[ D_{e_{\ell _1}} F\mathcal {N}D_{e_{\ell _2}} F \right] \langle e_{\ell _1}, e_{\ell _2} \rangle _{\dot{V}}\\&= \sum _{\ell _1, \ell _2} \mathbb {E} \left[ \left( \mathcal {N}^{1 / 2} D_{e_{\ell _1}} F \right) \mathcal {N}^{1 / 2} D_{e_{\ell _2}} F \right] \langle e_{\ell _1}, e_{\ell _2} \rangle _{\dot{V}}\\&= \sum _{\ell _1, \ell _2} \mathbb {E} \left[ D_{e_{\ell _1}} ((\mathcal {N}- 1)^{1 / 2} \boldsymbol{1}_{\mathcal {N} \geqslant 1} F) D_{e_{\ell _2}} ((\mathcal {N}- 1)^{1 / 2} \boldsymbol{1}_{\mathcal {N} \geqslant 1} F) \right] \langle e_{\ell _1}, e_{\ell _2} \rangle _{\dot{V}}\\&= \mathbb {E} \left[ \left\| D (\mathcal {N}- 1)^{1 / 2} \boldsymbol{1}_{\mathcal {N} \geqslant 1} F \right\| _{\dot{V}}^2 \right] . \end{aligned}$$

### Lemma A.3

Let $\rho \in L (V, V)\cap L(H, H)$. The tensorization $\rho ^{\otimes 2}$ of $\rho$ satisfies the following bound:

$$ \Vert \rho ^{\otimes 2} \varphi \Vert _{V \otimes _s H} \leqslant \Vert \rho \Vert _{L (V, V)} \Vert \rho \Vert _{L (H, H)} \Vert \varphi \Vert _{V \otimes _s H}, \qquad \varphi \in V \otimes _s H. $$

### Proof

$$\begin{aligned} \Vert \rho ^{\otimes 2} \varphi \Vert _{V \otimes _s H}^2&= \sum _k \Vert (\rho ^{\otimes 2} \varphi ) (\cdot , e_k) \Vert _V^2\\&\leqslant \Vert \rho \Vert _{L (V, V)}^2 \sum _k \Vert ((\operatorname {id} \otimes \rho ) \varphi ) (\cdot , e_k) \Vert _V^2\\&\leqslant \Vert \rho \Vert _{L (V, V)}^2 \Vert \rho \Vert _{L (H, H)}^2 \sum _k \Vert \varphi (\cdot , e_k) \Vert _V^2\\&= \Vert \varphi \Vert _{V \otimes _s H}^2 . \end{aligned}$$

$$\square $$

### Lemma A.4

If *S* is a core for *A*, then ${\mathcal {C}}$ is a core for ${\mathcal {L}}_0$.

### Proof

Let $F \in \mathcal {D} (\mathcal {L}_0)$ and let $(T_t)_{t \geqslant 0}$ be the semigroup generated by $\mathcal {L}_0$. The construction in Section 2 of [34] gives for $G (\eta ) = \Psi (\eta (g)) \in \mathcal {C}$ the Mehler formula

77 $$\begin{aligned} T_t G (\eta )&= \int G \left( e^{- t A} \eta + \sqrt{1 - e^{- 2 t A}} \zeta \right) \mu (\textrm{d}\zeta ) \nonumber \\&:=\int \Psi \left( \eta (e^{- t A} g) + \zeta \left( \sqrt{1 - e^{- 2 t A}} g \right) \right) \mu (\textrm{d}\zeta ), \end{aligned}$$

where $(e^{- t A})_{t \geqslant 0}$ is the semigroup generated by *A*. In the next steps, we build approximations in $\mathcal {C}$ that converge to *F* in graph norm.

1. Approximation of *F* by $A_t F$: Consider $A_t F :=\frac{1}{t} \int _0^t T_s F \textrm{d}s$, which converges to *F* in graph norm as $t \rightarrow 0$: Indeed, $A_t F \rightarrow F$ in $L^2 (\mu )$ by strong continuity of $(T_t)_{t \ge 0}$ and
  $$ \Vert \mathcal {L}_0 A_t F -\mathcal {L}_0 F \Vert = \left\| \frac{1}{t} (T_t F - F) -\mathcal {L}_0 F \right\| \rightarrow 0. $$
2. Approximation of $A_t F$ by $A_t F^N \in A_t \mathcal {C}$: Since $\mathcal {C}$ is dense in $\mathcal {H}^1_0$ by definition, we can find $(F^N)_N \subset \mathcal {C}$ with $\Vert F^N - F \Vert _{\mathcal {H}^1_0} \rightarrow 0$. We claim that $(A_t F^N)$ converges to $A_t F$ in graph norm as $N\rightarrow \infty$, for all $t > 0$. Indeed, $\Vert A_t F^N - A_t F \Vert _{\mathcal {H}^0_0} \rightarrow 0$ for all $t > 0$ because $A_t$ is a contraction, and also
  $$ \Vert \mathcal {L}_0 A_t F^N -\mathcal {L}_0 A_t F \Vert = \left\| \frac{1}{t} (T_t F^N - F^N) - \frac{1}{t} (T_t F - F) \right\| \rightarrow 0. $$
3. Approximation of $A_t F^N$ by $A^{(m)}_t F^N \in \mathcal {C} (D (A))$: For $t \geqslant 0$ and $G \in \mathcal {C}$ of the form $G(\eta )=\Psi (\eta (g))$ the Mehler formula shows that $T_t G(\eta ) = \Phi (\eta (e^{- t A} g))$ with the polynomial
  $$ \Phi (x) = \int \Psi \left( x + \zeta \left( \sqrt{1 - e^{- 2 t A}} g \right) \right) \mu (\textrm{d}\zeta ). $$
  Moreover, $e^{- t A} S \subset e^{- t A} D (A) \subset D (A)$, where *D*(*A*) is the domain of *A*. Therefore,
  $$\begin{aligned}&T_t \mathcal {C} \subset \mathcal {C} (\mathcal {D} (A)) :=\left\{ G \in L^2 (\Omega ) : G (\eta ) = \Phi (\eta (f)), f \in \mathcal {D} (A)^m,\right. \\&\left. \quad \Phi : \mathbb {R}^m \rightarrow \mathbb {R} \text { polynomial}, m \in \mathbb {N} \right\} . \end{aligned}$$
  Now consider the Riemann sum approximation of $A_t$:
  $$ A^{(m)}_t :=\frac{1}{m} \sum _{k = 0}^{m - 1} T_{\frac{k t}{m}} . $$
  Then $A^{(m)}_t F^N \in \mathcal {C} (D (A))$ for all $m, N \in \mathbb {N}$, and we claim that $A^{(m)}_t F^N \rightarrow A_t F^N$ in graph norm:
  $$ \Vert A^{(m)}_t F^N - A_t F^N \Vert \leqslant \frac{1}{t} \sum _{k = 0}^{m - 1} \int _{\frac{k t}{m}}^{\frac{(k + 1) t}{m}} \left\| \left( T_{\frac{k t}{m}} - T_s \right) F^N \right\| \textrm{d}s \rightarrow 0, \qquad m \rightarrow \infty , $$
  and since $F^N \in \mathcal {D} (\mathcal {L}_0)$ also
  $$ \Vert \mathcal {L}_0 A^{(m)}_t F^N -\mathcal {L}_0 A_t F^N \Vert = \Vert A^{(m)}_t \mathcal {L}_0 F^N - A_t \mathcal {L}_0 F^N \Vert \rightarrow 0, \qquad m \rightarrow \infty . $$
4. Approximation of $A^{(m)}_t F^N$ by $\mathcal {C}$: Since $A^{(m)}_t F^N \in \mathcal {C} (\mathcal {D} (A))$, there exist $k \in \mathbb {N}$, a polynomial $\Psi : \mathbb {R}^k \rightarrow \mathbb {R}$, and $g \in D (A)^k$ such that $A^{(m)}_t F^N (\eta ) = \Psi (\eta (g))$. Since *S* is a core for *A*, there exist $(g^i)_{i \in \mathbb {N}} \subset S^k$ with $g^i \rightarrow g$ and $A g^i \rightarrow A g$ in *H*. Then $(\Psi (\eta (g^i)))_i \in \mathcal {C}$ converges in graph norm to $\Psi (\eta (g)) = A^{(m)}_t F^N (\eta )$ as $i\rightarrow \infty$: The converge in $L^2 (\mu )$ is clear, and
  $$\begin{aligned} \mathcal {L}_0 \Psi (\eta (g^i))&= \sum _{\ell = 1}^k \partial _{\ell } \Psi (\eta (g^i)) (\eta (- A g^i_{\ell })) + \sum _{\ell _1, \ell _2 = 1}^k \partial _{\ell _1, \ell _2} \Psi (\eta (g^i)) \langle - A g^i_{\ell _1}, g^i_{\ell _2} \rangle \\&\rightarrow \sum _{\ell = 1}^k \partial _{\ell } \Psi (\eta (g)) (\eta (- A g_{\ell })) + \sum _{\ell _1, \ell _2 = 1}^k \partial _{\ell _1, \ell _2} \Psi (\eta (g)) \langle - A g_{\ell _1}, g_{\ell _2} \rangle \\&=\mathcal {L}_0 A^{(m)}_t F^N (\eta ). \end{aligned}$$

The combination of Steps 1.-4. shows that *F* can be approximated by elements of $\mathcal {C}$ in graph norm, so $\mathcal {C}$ is a core for $\mathcal {D} (\mathcal {L}_0)$. $\square$

### Lemma A.5

(Bounds for $\rho _N$ of Example 4.1) Let $0 \leqslant \alpha \leqslant \beta \leqslant 1$ and let *D* and $\rho _N$ be as in Example 4.1. Then

$$\begin{aligned} \Vert \rho _N v \Vert _{H^{\beta }}&\lesssim N^{\beta - \alpha } \Vert v \Vert _{H^{\alpha }}, \\ \Vert \rho _N v - v \Vert _{H^{\alpha }}&\lesssim N^{- (\beta - \alpha )} \Vert v \Vert _{H^{\beta }}, \end{aligned}$$

and the same bounds also hold if we replace $H^\alpha$ and $H^\beta$ by their homogeneous counterparts $\dot{H}^\alpha$ and $\dot{H}^\beta$.

### Remark A.6

Before going to the proof, we stress that the specific form of $I^N_x$ is never used in the arguments, and we only need that $y \in I^N_x$ forces $|{p_N(x)}-y| \leqslant 1/N$ and that $|I^N_x \cap D| = 1$ for all $x\in D$.

### Proof

We use interpolation arguments to reduce to the special cases $\alpha , \beta \in \{ 0, 1 \}$. We focus on the inhomogeneous estimates, the homogeneous estimate follows from the same arguments, since along the way we show $\Vert \rho _N v\Vert _{\dot{H}^1} \lesssim \Vert v\Vert _{\dot{H}^1}$ and $\Vert \rho _N v - v\Vert _{L^2} \lesssim \Vert v\Vert _{\dot{H}^1}$.

1. Estimates for $\Vert \rho _N v \Vert _{H^{\beta }}^2$: For $\alpha = \beta = 0$ we use that $N \boldsymbol{1}_{I^N_x}$ defines a probability measure for each $x \in D$ and we have $y \in I^N_x$ only if $|{p_N(x)} - y | < \frac{1}{N}$, and thus by Jensen’s inequality and Fubini’s theorem
  78 $$\begin{aligned} \Vert \rho _N v \Vert _{L^2}^2&= \int _D \left( \int _D N \boldsymbol{1}_{(p_N(x),p_N(x)+1/N)}(y) v (y) \textrm{d}y \right) ^2 \textrm{d}x\nonumber \\&\leqslant \int _D \int _D N \boldsymbol{1}_{(p_N(x),p_N(x)+1/N)}(y) |v (y)|^2 \textrm{d}y \textrm{d}x\nonumber \\&\leqslant N\int _D \int _{p_N(D)}\boldsymbol{1}_{(x,x+1/N)}(y)\frac{1}{p_N'(x)} \textrm{d}x| v (y) |^2\textrm{d}y\nonumber \\&\lesssim \Vert v\Vert _{L^2}^2, \end{aligned}$$
  where we performed a change of variables in the third step and then used (70). Using an implicit smooth approximation of *v* and $\iota _N$, the convolution identity (71), a change of variables and Young’s convolution inequality gives
  79 $$\begin{aligned} \Vert \partial _x \rho _Nv\Vert _{L^2}^2&=\int _D|(v*\partial _x\iota _{N})(p_N(x))p_N'(x)|^2\,\textrm{d}x\nonumber \\&\lesssim N^2 \int _{D}|v(p_N(x)+1/N)-v(p_N(x))|^2\,\textrm{d}x\nonumber \\&\lesssim N^2 \Vert v\Vert _{L^2}^2\,\textrm{d}x, \end{aligned}$$
  and so
  80 $$\begin{aligned} \Vert \partial _x \rho _N v \Vert _{L^2}^2 \lesssim N^2 \Vert v \Vert _{L^2}^2 \qquad \Rightarrow \qquad \Vert \rho _N v \Vert _{H^1} \lesssim N \Vert v \Vert _{L^2} . \end{aligned}$$
  By repeating the same computation as in (79) but putting the derivative on *v*, it follows that
  $$\begin{aligned} \Vert \rho _N v \Vert _{H^1} \lesssim \Vert v \Vert _{H^1} . \end{aligned}$$
  Let $X_0 = X_1 = L^2$ and $Y_0 = L^2$, $Y_1 = H^1$, so that $\Vert \rho _N \Vert _{L (X_0, Y_0)} \lesssim 1$ and $\Vert \rho _N \Vert _{L (X_1, Y_1)} \lesssim N$. For $\theta = \beta$ we have $[X_0, X_1]_{\theta } = L^2$ and $[Y_0, Y_1]_{\theta } = H^{\beta }$, so the interpolation theorem yields for some $C > 0$ that does not depend on *N*
  81 $$\begin{aligned} \Vert \rho _N \Vert _{L (L^2, H^{\beta })} \leqslant C^{1 - \theta } (C N)^{\theta } \simeq N^{\beta } . \end{aligned}$$
  Interpolation with $X_0 = Y_0 = L^2$ and $X_1 = Y_1 = H^1$ gives $[X_0, X_1]_{\theta } = [Y_0, Y_1]_{\theta } = H^{\beta }$, and thus
  82 $$\begin{aligned} \Vert \rho _N \Vert _{L (H^{\beta }, H^{\beta })} \lesssim 1. \end{aligned}$$
  Now we interpolate (81) and (82) with $\theta = \frac{\beta - \alpha }{\beta }$ and $X_0 = L^2$, $Y_0 = H^{\beta }$ and $X_1 = Y_1 = H^{\beta }$, so that $[X_0, X_1]_{\theta } = H^{\beta (1 - \theta )} = H^{\alpha }$ and $[Y_0, Y_1]_{\theta } = H^{\beta }$ and
  $$ \Vert \rho _N \Vert _{L (H^{\beta }, H^{\beta })} \lesssim N^{\beta \theta } = N^{\beta - \alpha } . $$
2. Estimates for $\Vert \rho _N v - v \Vert _{H^{\alpha }}^2$: For $\alpha = \beta = 0$ and $\alpha = \beta = 1$ we simply use the triangle inequality. For $\alpha = 0$ and $\beta = 1$ we bound, writing $(x, y) = (y, x)$ if $y < x$,
  $$\begin{aligned} \Vert \rho _N v - v \Vert _{L^2}^2&= \int _D \left( \int _D N \boldsymbol{1}_{I^N_x} (y) (v (y) - v (x)) \textrm{d}y \right) ^2 \textrm{d}x\\&\leqslant \int _D \int _D N \boldsymbol{1}_{I^N_x} (y) \left( \int _x^y \partial _z v (z) \textrm{d}z \right) ^2 \textrm{d}y \textrm{d}x\\&\leqslant \int _D \int _D \int _D N | y - x |\boldsymbol{1}_{I^N_x} (y) \boldsymbol{1}_{(x, y)} (z) | \partial _z v (z) |^2 \textrm{d}z \textrm{d}y \textrm{d}x\\&\leqslant \int _D \int _D \int _D N | y - x | \boldsymbol{1}_{| p_N(x) - y | \leqslant \frac{1}{N}} \boldsymbol{1}_{| z - p_N(x) | \leqslant \frac{2}{N}} | \partial _z v (z) |^2 \textrm{d}z \textrm{d}y \textrm{d}x\\&\lesssim N^{- 2} \Vert \partial _z v \Vert _{L^2}^2, \end{aligned}$$
  where in the penultimate and last step we used that $p_N(x)\leqslant x \leqslant p_N(x)+2/N$, which implies that $|p_N(x)-x|\leqslant 2/N$. Thus, $\Vert \rho _N v - v \Vert _{L^2} \lesssim N^{- 1} \Vert v \Vert _{H^1}$. Interpolation with $X_0 = Y_0 = L^2$ and $X_1 = H^1$, $Y_1 = L^2$ and $1 - \theta = \beta$ yields
  83 $$\begin{aligned} \Vert \rho _N - \operatorname {id} \Vert _{L (H^{\beta }, L^2)} \lesssim N^{- \beta } . \end{aligned}$$
  Taking instead $Y_1 = H^1$, we get
  84 $$\begin{aligned} \Vert \rho _N - \operatorname {id} \Vert _{L (H^{\beta }, H^{\beta })} \lesssim 1. \end{aligned}$$
  Interpolating (83) and (84) with $\theta = \frac{\alpha }{\beta }$, we finally obtain
  $$ \Vert \rho _N - \operatorname {id} \Vert _{L (H^{\beta }, H^{\alpha })} \lesssim N^{- \beta (1 - \theta )} = N^{- (\beta - \alpha )} . $$
  $$\square $$

### Lemma A.7

(Trace operator on Sobolev spaces) Let $D \subset \mathbb {R}^d$ be a Lipschitz domain and let $s > \frac{d}{2}$. Then the trace

$$\begin{aligned} T \varphi (x) :=\varphi (x, x), \qquad x \in D, \end{aligned}$$

defines a bounded linear operator $T : H^s (D \times D) \rightarrow H^{s - d / 2} (D)$.

### Proof

According to [35, Ch. 4.1] we have

$$ \Vert T \varphi \Vert _{H^{s - d / 2} (D)} \simeq \inf \{ \Vert \psi \Vert _{H^{s - d / 2} (\mathbb {R}^d)} : \psi |_D = T \varphi \}, $$

and similarly for $\Vert \varphi \Vert _{H^s (D \times D)}$. If $\psi \in H^s (\mathbb {R}^{2 d})$ is such that $\psi |_{D \times D} = \varphi$, then $T \psi |_D = T \varphi$ and thus

$$ \Vert T \varphi \Vert _{H^{s - d / 2} (D)} \lesssim \inf \{ \Vert T \psi \Vert _{H^{s - d / 2} (\mathbb {R}^d)} : \psi |_{D \times D} = \varphi \} . $$

But on $\mathbb {R}^d$ we can access Fourier methods to compute $\mathcal {F} (T \psi ) (z) = \int _{\mathbb {R}^d} \mathcal {F} \psi (z - z', z') \textrm{d}z'$ and then

$$\begin{aligned}&\Vert T \psi \Vert _{H^{s - d / 2} (\mathbb {R}^d)}^2\\&= \int _{\mathbb {R}^d} (1 + | z |^2)^{s - \frac{d}{2}} | \mathcal {F} (T \psi ) (z) |^2 \textrm{d}z\\&\leqslant \int _{\mathbb {R}^d} (1 + | z |^2)^{s - \frac{d}{2}} \left( \int _{\mathbb {R}^d} (1 + | z - z' |^2 + | z' |^2)^{- s} \textrm{d}z' \right) \\&\quad \left( \int _{\mathbb {R}^d} (1 + | z - z' |^2 + | z' |^2)^s | \mathcal {F} \psi (z - z', z') |^2 \textrm{d}z' \right) \textrm{d}z\\&\lesssim \int _{\mathbb {R}^d} (1 + | z |^2)^{s - \frac{d}{2}} (1 + | z |^2)^{\frac{d}{2} - s} \int _{\mathbb {R}^d} (1 + | z - z' |^2 + | z' |^2)^s | \mathcal {F} \psi (z - z', z') |^2 \textrm{d}z' \textrm{d}z\\&= \Vert \psi \Vert _{H^s (\mathbb {R}^d \times \mathbb {R}^d)}, \end{aligned}$$

using $s > \frac{d}{2}$ in the fourth line to bound the integral $\int _{\mathbb {R}^d} (1 + | z - z' |^2 + | z' |^2)^{- s} \textrm{d}z'$. Thus, we have shown that

$$ \Vert T \varphi \Vert _{H^{s - d / 2} (D)} \lesssim \inf \{ \Vert \psi \Vert _{H^s (\mathbb {R}^d \times \mathbb {R}^d)} : \psi |_{D \times D} = \varphi \} = \Vert \varphi \Vert _{H^s (D \times D)} . $$

$$\square $$

### Lemma A.8

(Regularity of the derivative on Neumann Sobolev spaces) Consider for $\theta \in \mathbb {R}$ the Neumann Sobolev spaces

$$ H^{\theta } = \left\{ h = \sum _{k = 0}^{\infty } \cos (\pi k \cdot ) \hat{h} (k) : \Vert f \Vert _{H^{\theta }}^2 = \sum _{k = 0}^{\infty } (1 + | k |^2)^{\theta } | \hat{h} (k) |^2 < \infty \right\} , $$

and for $\theta < 0$ we interpret $h \in H^{\theta }$ as an element of the dual space of $H^{- \theta }$. Let $\theta \in \left( \frac{1}{2}, \frac{5}{2} \right)$ and $\delta > \frac{3}{2}$. Then the space derivative $\partial _x$ is a bounded linear operator from $H^{\theta }$ to $H^{\theta - \delta }$.

### Proof

We consider *h* of the form $h = \sum _{k = 0}^N \cos (\pi k \cdot ) \hat{h} (k)$ and derive an estimate that is uniform in *N*. The Fourier type coefficients of $\partial _x h$ are

$$\begin{aligned} | \widehat{\partial _x h} (k) |&= \left| \int _0^1 \cos (\pi k x) \sum _{\ell = 0}^N (- \pi \ell ) \sin (\pi \ell x) \hat{h} (\ell ) \textrm{d}x \right| \\&= \left| \sum _{\ell = 0}^N (- \pi \ell ) \hat{h} (\ell ) \int _0^1 \cos (\pi k x) \sin (\pi \ell x) \textrm{d}x \right| \\&= \left| \sum _{\ell = 0}^N (- \pi \ell ) \hat{h} (\ell ) \boldsymbol{1}_{\ell \ne k} \frac{\ell (1 - (- 1)^{k + \ell })}{\pi (\ell ^2 - k^2)} \right| \\&\lesssim \left( \sum _{\ell = 0}^N (1 + | \ell |^2)^{\theta } | \hat{h} (\ell ) |^2 \right) ^{1 / 2} \left( \sum _{\ell> 0, \ell \ne k} \frac{\ell ^{4 - 2 \theta }}{(\ell - k)^2 (\ell + k)^2} \right) ^{1 / 2}\\&\lesssim \Vert h \Vert _{H^{\theta }} \left( \sum _{\ell < k} \frac{\ell ^{4 - 2 \theta }}{(\ell - k)^2 k^2} + \sum _{\ell > k} \frac{\ell ^{2 - 2 \theta }}{(\ell - k)^2} \right) ^{1 / 2}\\&\lesssim \Vert h \Vert _{H^{\theta }} ((1 + | k |^2)^{1 - \theta })^{1 / 2}, \end{aligned}$$

where the last step follows by comparing the sums with integrals and using that $\theta \in \left( \frac{1}{2}, \frac{5}{2} \right)$. Therefore,

$$\begin{aligned} \Vert \partial _x h \Vert _{H^{\theta - \delta }}^2&= \sum _{k = 0}^{\infty } (1 + | k |^2)^{\theta - \delta } | \widehat{\partial _x h} (k) |^2\\&\lesssim \Vert h \Vert _{H^{\theta }}^2 \sum _{k = 0}^{\infty } (1 + | k |^2)^{\theta - \delta } (1 + | k |^2)^{1 - \theta }\\&= \Vert h \Vert _{H^{\theta }}^2 \sum _{k = 0}^{\infty } (1 + | k |^2)^{1 - \delta }, \end{aligned}$$

and the series on the right hand side is finite if $\delta > \frac{3}{2}$. $\square$

## Footnotes

1 Although Fourier methods can still be useful in concrete examples.
